# Marked Differences in Mucosal Immune Responses Induced in Ileal versus Jejunal Peyer’s Patches to *Mycobacterium avium* subsp. *paratuberculosis* Secreted Proteins following Targeted Enteric Infection in Young Calves

**DOI:** 10.1371/journal.pone.0158747

**Published:** 2016-07-07

**Authors:** Antonio Facciuolo, Patricia Gonzalez-Cano, Scott Napper, Philip J. Griebel, Lucy M. Mutharia

**Affiliations:** 1 Department of Molecular and Cellular Biology, University of Guelph, Guelph, ON, Canada; 2 VIDO-InterVac, University of Saskatchewan, Saskatoon, SK, Canada; 3 Department of Biochemistry, University of Saskatchewan, Saskatoon, SK, Canada; 4 School of Public Health, University of Saskatchewan, Saskatoon, SK, Canada; University of Minnesota, UNITED STATES

## Abstract

In cattle, *Mycobacterium avium* subsp. *paratuberculosis* infection is primarily mediated through M cells overlying Peyer’s patches (PP) in the ileum. The capacity of *M*. *avium* subsp. *paratuberculosis* to invade ileal PP (IPP) versus discrete PP in the jejunum (JPP) and subsequent differences in mucosal immune responses were investigated. Intestinal segments were surgically prepared in both mid-jejunum, containing two JPPs, and in terminal small intestine containing continuous IPP. *M*. *avium* subsp. *paratuberculosis* (10^9^ CFU) was injected into the lumen of half of each intestinal segment when calves were 10–14 days-old and infection confirmed 1–2 months later by PCR and immunohistochemistry. Thirteen recombinant *M*. *avium* subsp. *paratuberculosis* proteins, previously identified as immunogenic, were used to analyze pathogen-specific B- and T-cell responses in PP and mesenteric lymph nodes. IgA plasma cell responses to 9 of 13 recombinant proteins were detected in JPP but not in IPP. Secretory IgA reacting in ELISA with 9 of the 13 recombinant proteins was detected in luminal contents from both jejunal and ileal segments. These observations support the conclusion that pathogen-specific IgA B cells were induced in JPP but not IPP early after a primary infection. The presence of secretory IgA in intestinal contents is consistent with dissemination of IgA plasma cells from the identified mucosa-associated immune induction sites. This is the first direct evidence for *M*. *avium* subsp. *paratuberculosis* uptake by bovine JPP and for local induction of pathogen-specific IgA plasma cell responses after enteric infection. We also provide evidence that bacterial invasion of IPP, a primary B lymphoid tissue, provides a novel strategy to evade induction of mucosal immune responses. Over 60% of PPs in the newborn calf small intestine is primary lymphoid tissue, which has significant implications when designing oral vaccines or diagnostic tests to detect early *M*. *avium* subsp. *paratuberculosis* infections.

## Introduction

*Mycobacterium avium* subspecies *paratuberculosis* is the etiological agent of Johne’s disease, a chronic enteric infection of ruminant animals that occurs worldwide and has significant impacts on animal production [1]. Young ruminants, such as newborn calves, are the most susceptible to *M*. *avium* subsp. *paratuberculosis* infections through fecal-oral transmission [2]. In calves and goat kids, M cells overlying Peyer’s Patches (PP) in the terminal small intestine were confirmed to mediate *M*. *avium* subsp. *paratuberculosis* uptake across the intestinal epithelium barrier with subsequent invasion of underlying subepithelial macrophages [3, 4]. *M*. *avium* subsp. *paratuberculosis* then utilizes a variety of mechanisms to subvert innate and acquired immune defenses and establish persistent intracellular infections within macrophages [5]. The asymptomatic nature of *M*. *avium* subsp. *paratuberculosis* infections results in subclinical disease that may persist for years, during which time transmission occurs through intermittent shedding of bacilli in feces and milk. Infected adult cows, both with and without clinical disease, develop paratuberculosis-associated lesions primarily located in the terminal small intestine but lesions may be disseminated throughout the small intestine and associated lymphoid tissues [6]. Progression of Johne’s disease is also associated with a gradual decline in the total number of CD4^+^ T cell located within the lamina propria of the infected ileum [7].

Characterizing the pathobiological events during early stages of *M*. *avium* subsp. *paratuberculosis* infection is difficult in naturally infected animals due to the prolonged absence of clinical symptoms and a lack of biological markers that identify recently infected animals. A variety of infection models have, however, been developed in young calves to study early host-pathogen interactions [8–12]. Tonsillar crypt deposition of *M*. *avium* subsp. *paratuberculosis* in 2–5 week old calves demonstrated that antibody and T cell-mediated immune responses were not detected in blood until 4 and 6 months post-infection (PI), respectively [8]. Western blot analysis, however, revealed that serum antibodies specific to two *M*. *avium* subsp. *paratuberculosis* proteins could be detected within two weeks PI. Cannulation of intestinal lymphatics has also been used to analyze changes in the lymphoid cells emigrating from the terminal small intestine of calves infected at 2 months of age [9]. Temporary occlusion of the intestinal lumen has been used to target *M*. *avium* subsp. *paratuberculosis* infection to the terminal small intestine of 2–3 week-old calves and subsequent analyses revealed altered cytokine gene expression in the terminal small intestine and draining mesenteric lymph node (MLN) over the course of 9 months [10]. More recently, surgically isolated intestinal segments have been prepared in 10–14 day old calves and used to restrict *M*. *avium* subsp. *paratuberculosis* infection to a localized region of the small intestine for as long as 9–11 months PI [12]. This model was used to analyze *M*. *avium* subsp. *paratuberculosis*-specific changes in the phenotype and function of local mucosal myeloid and lymphoid cell populations.

The majority of enteric *M*. *avium* subsp. *paratuberculosis* infection models developed for studying host-pathogen interactions have targeted infection to the continuous or ileal PP located in the terminal small intestine of young calves. These studies either focused on host responses within hours of infection [3, 11] or analyzed host responses within weeks to months PI [9, 10, 12]. However, the question remains whether there are unique immunological responses following *M*. *avium* subsp. *paratuberculosis* uptake by either the discrete PP, located throughout the jejunum (JPP), versus the continuous ileal PP (IPP) located in the terminal small intestine. Sampling of *M*. *avium* subsp. *paratuberculosis* by JPP has been confirmed for lambs [13] but has not been evaluated in calves. This question may have broad implications for the pathogenesis of *M*. *avium* subsp. *paratuberculosis* infection when the marked functional differences between JPP versus IPP are considered. JPP function primarily as mucosal immune induction sites [14, 15] for the production of IgA plasma cells in the presence of numerous CD4^+^ follicular T cells [16, 17]. In contrast, the IPP is characterized by a paucity of CD4^+^ follicular T cells and follicular B cells fail to undergo isotype-switching to IgA or IgG [16, 17]. Analysis of activation-induced cytidine deaminase (AID) expression in bovine IPP further supports the conclusion that this mucosa-associated lymphoid tissue (MALT) functions as a site for antigen-independent expansion of the primary antibody repertoire [18]. This is consistent with results from a previous study where ovine IPP failed to develop detectable IgA plasma cell response following enteric vaccination [15]. In contrast to the phenotypic differences in lymphoid cell populations, comparative analyses of myeloid cell populations have shown that antigen-presenting cells are abundant and phenotypically similar in bovine JPP and IPP compartments such as the lamina propria and lymphoid follicles [19].

It is not known what the immunopathological consequences to *M*. *avium* subsp. *paratuberculosis* infection may be for this functional dichotomy observed for the immune induction sites located within the lymphoid follicles of JPP versus IPP. It is also not known what *M*. *avium* subsp. *paratuberculosis* proteins may be involved in the induction of mucosal immune responses during the early phase of infection, despite evidence that at least two unknown *M*. *avium* subsp. *paratuberculosis* proteins may be recognized within two weeks PI [8]. Defining *M*. *avium* subsp. *paratuberculosis* proteins that are immunogenic during the early, acute phase of infection may have significant value for developing diagnostic tests and identifying vaccine candidates that can alter the course of infection. The objective of this study was to target *M*. *avium* subsp. *paratuberculosis* infection to both JPP and IPP in 10–14 day-old calves and determine if significant regional differences in mucosal immunity were observed within one to two months PI. Surgically isolated intestinal segments, containing either two JPP or a continuous IPP, were used to target *M*. *avium* subsp. *paratuberculosis* infection to both MALTs and facilitate a direct comparison of local mucosal immune responses [12]. Thirteen recombinant *M*. *avium* subsp. *paratuberculosis* proteins, previously identified as immunogenic in Western blots with sera from *M*. *avium* subsp. *paratuberculosis*-infected cows [20], were used to monitor the magnitude and diversity of regional mucosal immune responses induced during the early phase of *M*. *avium* subsp. *paratuberculosis* infection. We observed marked regional differences in enteric mucosal immune responses to *M*. *avium* subsp. *paratuberculosis* infection, and identified a subset of bacterial proteins that induce both mucosal and systemic B cell responses during the acute phase of infection. The implications of bacterial invasion of a primary gut-associated lymphoid tissue are also discussed within the context of immune evasion and establishing a persistent mycobacterial infection.

## Results

### Clinical observation of calves

Daily physical examination of calves post-surgery by animal health technicians revealed no changes in body temperature (> 1°C) or signs of abdominal pain or discomfort. Further, no changes in feed intake or fecal consistency were observed throughout the one to two month PI period.

### Gross anatomy and histology of intestinal segments

Isolated intestinal segments were harvested at either one or two months PI. Examination of the serosal surface of mid-jejunal and terminal small intestinal segments revealed no grossly visible abnormalities, adhesions, or discolouration when compared to the adjacent small intestine (S1A and S1B Fig). Each compartment within the segments remained distinct with occlusion of the mucosal lumen at the junction between compartments. Accumulation of intestinal contents was visible in each compartment. This material arises due to the continuous epithelial cell proliferation and turnover in addition to the production of mucus from goblet cells. This material preferentially accumulated at the distal end of each compartment which is consistent with peristaltic activity moving intestinal contents in an aboral direction. Examination of the mucosal surfaces revealed no grossly visible abnormalities, with no evidence of mucosal erosion or discolouration relative to the adjacent intestine. The JPP and IPP were visible with clearly demarcated borders (S1C and S1D Fig). Microscopic evaluation of H&E stained tissue sections revealed intact villi with follicle-associated epithelium overlying the dome region of lymphoid follicles (S1E and S1F Fig). Numerous lymphoid follicles were visible in the submucosa with distinct structural differences between the JPP and IPP (S1E and S1F Fig). IPP collected from terminal small intestinal segments were characterized by numerous, closely packed submucosal lymphoid follicles with small interfollicular accumulations of cells. In contrast, the JPP collected from mid-jejunal segment, while also characterized by numerous submucosal lymphoid follicles, were frequently separated by extensive accumulations of interfollicular cells. No cellular aggregates were observed in the lamina propria of either the *M*. *avium* subsp. *paratuberculosis*-infected mid-jejunal or terminal small intestinal compartments.

**Fig 1:**
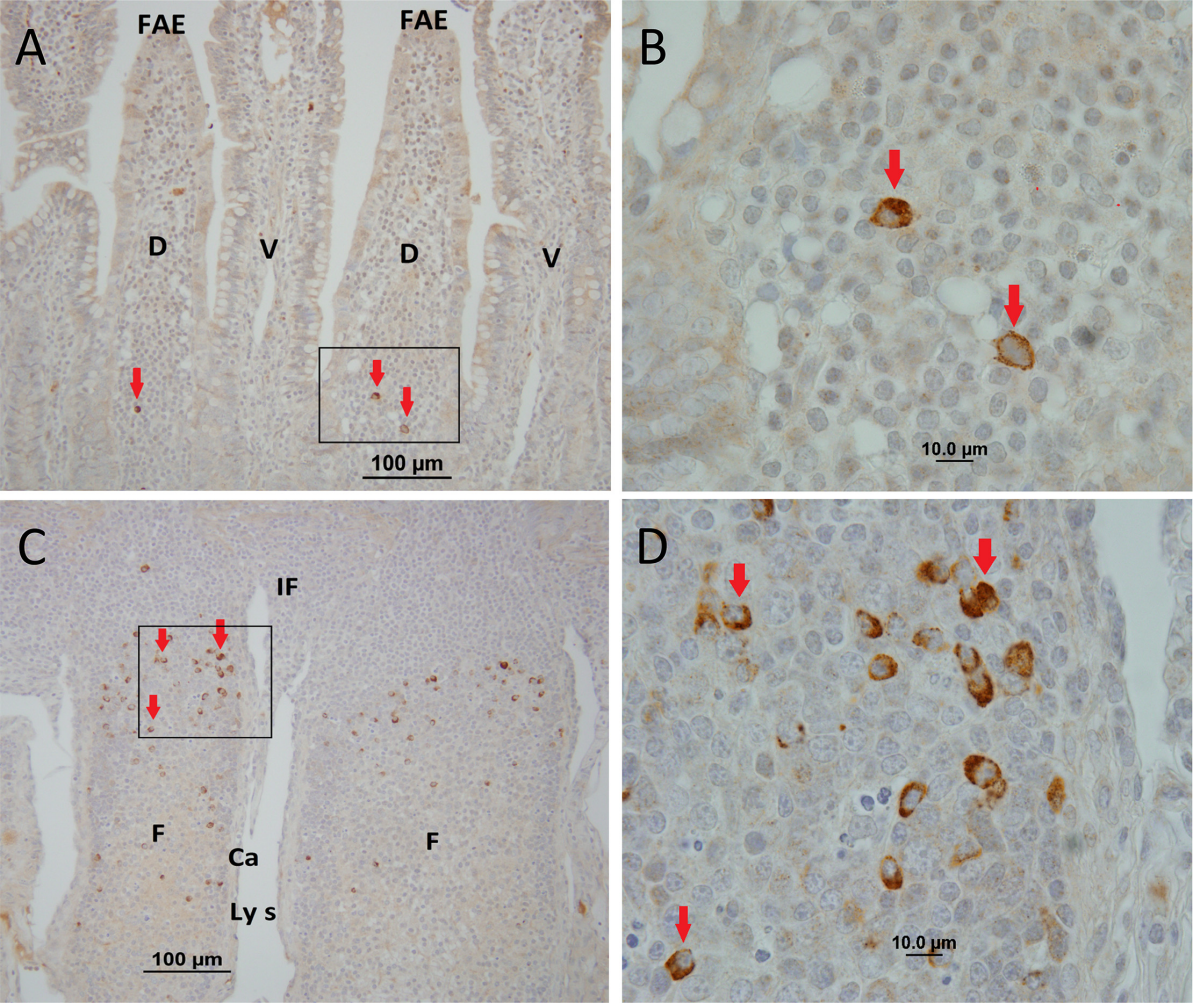
Representative immunohistochemical staining of tissue samples collected from surgically isolated terminal small intestinal segments infected with *M*. *avium* subsp. *paratuberculosis* at one to two months PI. *M*. *avium* subsp. *paratuberculosis* staining (brown colour and arrowheads) was observed in the dome regions (A, B), and was most abundant within cells of the lymphoid follicles in the submucosa (C, D). FAE, follicle-associated epithelium; D, dome region; V, villus; F, follicle; IF, interfollicular region; Ls, lymphatic sinus; Ca, capsule. Magnification is at x20 (A, C) and x100 (B, D).

**Fig 2:**
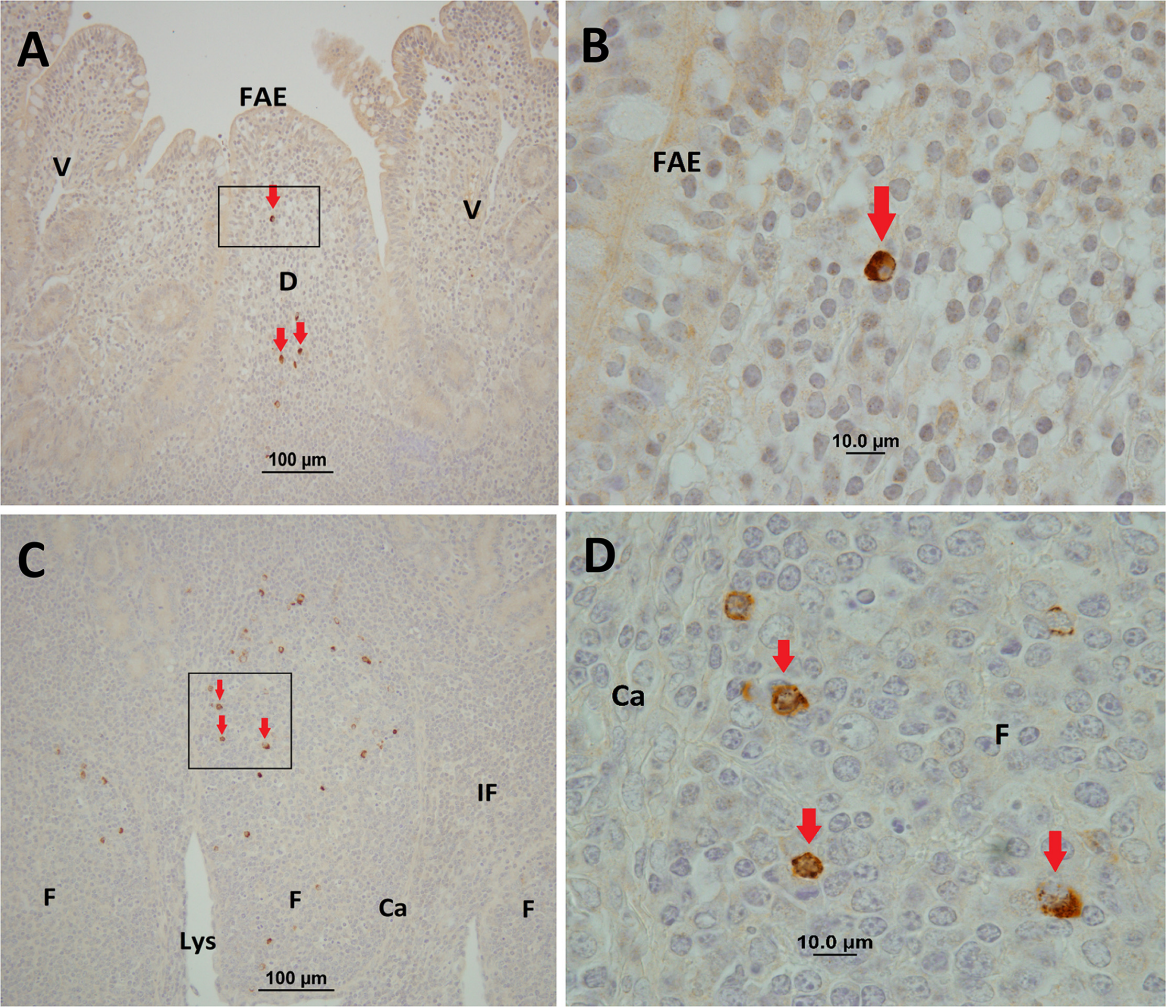
Representative immunohistochemical staining of tissue samples collected from surgically isolated mid-jejunal intestinal segments infected with *M*. *avium* subsp. *paratuberculosis* at one to two months PI. *M*. *avium* subsp. *paratuberculosis* antigen staining (brown colour and arrowheads) was observed in the dome regions (A, B), and was most abundant within cells of the lymphoid follicles in the submucosa (C, D). *M*. *avium* subsp. *paratuberculosis* staining was not observed in the interfollicular regions. FAE, follicle-associated epithelium; D, dome region; V, villus; F, follicle; IF, interfollicular region; Ls, lymphatic sinus; Ca, capsule. Magnification is at x20 (A, C) and x100 (B, D).

*M*. *avium* subsp. *paratuberculosis* cells and its proteins were detected in tissues by IHC staining using polyclonal antisera. IHC staining revealed *M*. *avium* subsp. *paratuberculosis* was located primarily within the follicle-associated dome regions and within cells of the underlying lymphoid follicles in both IPP (Fig 1) and JPP (Fig 2). In contrast, no visible IHC staining was observed in the interfollicular regions of *M*. *avium* subsp. *paratuberculosis*-infected IPP (Fig 1) and JPP (Fig 2). The frequency and intensity of IHC stained cells was not uniform among individual lymphoid follicles. Visible cellular staining was detected in 49.0% (range 27.7–67.4%, n = 7) of IPP follicles versus 71.2% (range 34.5–94.7%, n = 7) of JPP follicles in *M*. *avium* subsp. *paratuberculosis*-infected segments. IHC staining of tissue sections from adjacent uninfected control compartments in both the mid-jejunal and terminal small intestinal segments did not reveal visible staining of cells for *M*. *avium* subsp. *paratuberculosis* (S2 Fig). IHC staining of draining MLNs revealed *M*. *avium* subsp. *paratuberculosis* in the cortex and medulla of 4 of the 7 MLNs draining the terminal small intestinal segments, and in 6 of the 7 MLNs draining mid-jejunal segments (data not shown). PCR was used to determine the presence or absence of *M*. *avium* subsp. *paratuberculosis*-specific DNA in infected and uninfected compartments within each segment. *M*. *avium* subsp. *paratuberculosis* DNA was detected in tissue sections from 6 of the 7 terminal small intestinal and all 7 mid-jejunal compartments injected with *M*. *avium* subsp. *paratuberculosis*. Among the compartments injected with PBS, *M*. *avium* subsp. *paratuberculosis*-specific DNA was detected in two terminal small intestinal compartments (calf #5 and #9; Table 1), and two mid-jejunal compartments (calf #8 and #9; Table 2). Consequently, data collected from these calves was excluded from the analysis of mucosal immune responses since the specificity of this analysis was based on a comparison of *M*. *avium* subsp. *paratuberculosis*-infected versus the adjacent uninfected compartment. Thus, only data from calves 6, 7, and 10 were used for the comparative analyses of mucosal immune responses in PP.

**Table 1:** PCR and IHC Detection of *M*. *avium* subsp. *paratuberculosis* in uninfected terminal small intestine segments (IPP-C), adjacent infected segments (IPP-M), and mesenteric lymph nodes (LN).

Calf	Infection (Mon)	PCR^1^		IHC^2^		
		IPP-C^3^	IPP-M^3^	IPP-C	IPP-M	LN
**4**	2.5	–	–	+	+	+
**5**	2	+	+	+ Tiss	+ Tiss	+
**6**	2	–	+	–	+	–
**7**	2	–	+	–	+	–
**8**	2	–	+	+	+	–
**9**	1	+	+	+	+	+
**10**	1	–	+	–	+ Cont	+

^1^PCR amplification using VetAlert™ Johne’s Real-Time PCR

^2^ *M*. *avium* subsp. *paratuberculosis* was detected using rabbit polyclonal antisera that may cross-react with other mycobacterial

^3^Continuous PP and draining MLN tissue were combined on the same slide used for the PCR reaction

Tiss = Tissue section with no visible intestinal contents attached to the mucosa

Cont = Intestinal contents on the slide without intestinal tissue

**Table 2:** PCR and IHC Detection of *M*. *avium* subsp. *paratuberculosis* in uninfected mid-jejunal segments (JPP-C), adjacent infected segments (JPP-M), and mesenteric lymph nodes (LN).

Calf	Infection (Mon)	PCR^1^		IHC^2^			
		JPP-C^3^	JPP-M^3^	JPP-C	LN	JPP-M	LN
**4**	2.5	–	+	–	–	+	+
**5**	2	–	+	–	+	+ Tiss	+
**6**	2	–	+	–	+	+	–
**7**	2	–	+	–	–	+	–
**8**	2	+	+	–	–	No Data	+
**9**	1	+	+	+	–	+	+
**10**	1	–	+	–	+	+ Tiss	+

^1^PCR amplification using VetAlert™ Johne’s Real-Time PCR

^2^ *M*. *avium* subsp. *paratuberculosis* was detected using rabbit polyclonal antisera that may cross-react with other mycobacterial species

^3^Discrete PP and draining MLN tissue were combined on the same slide used for the PCR reaction

Tiss = Tissue section with no visible intestinal contents attached to the mucosa

No Data: PP tissue absent from tissue collected in formalin

### B cell responses in JPP and IPP

**Fig 3:**
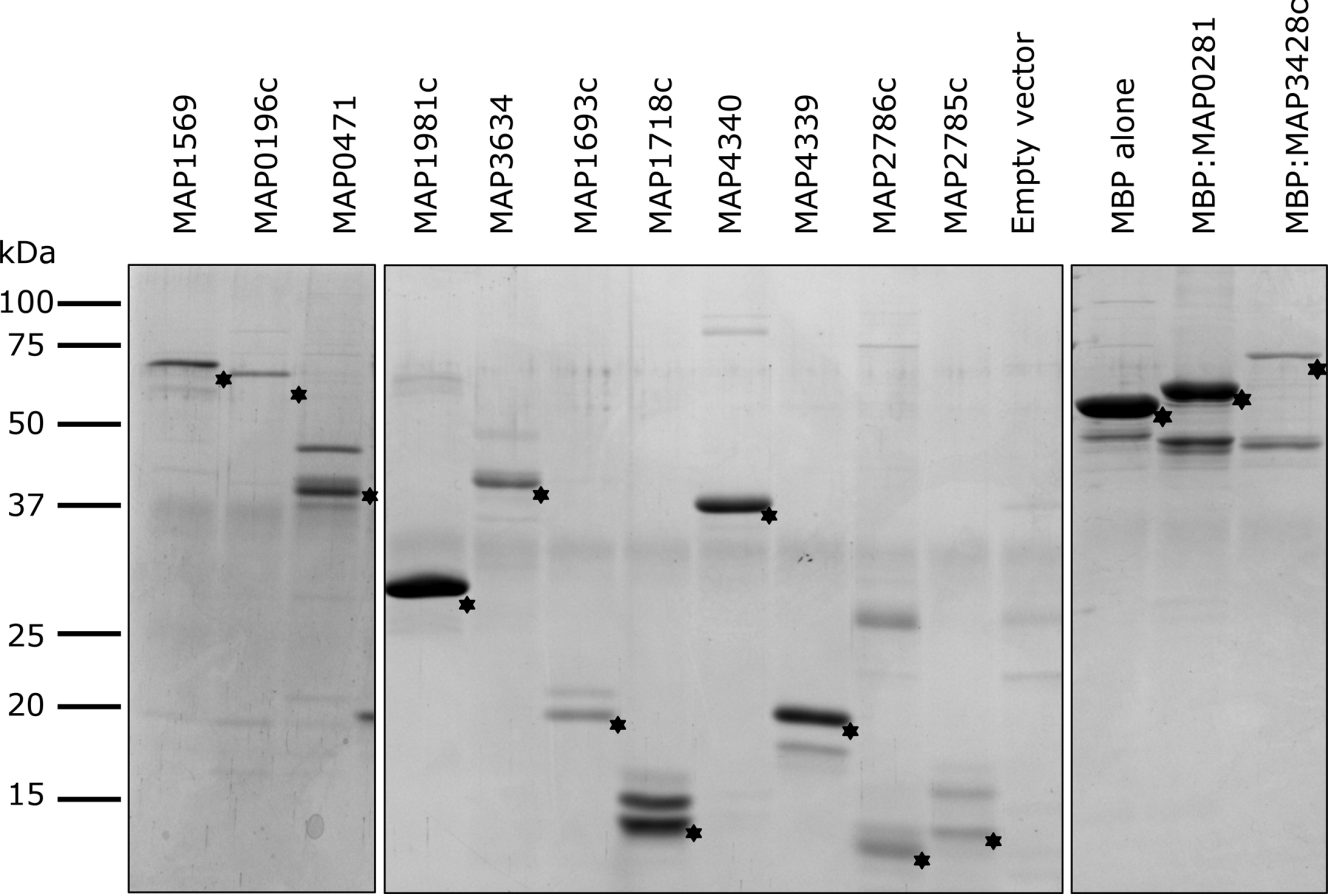
Recombinant *Mycobacterium avium* subsp. *paratuberculosis* proteins expressed in *E*. *coli* and purified by affinity chromatography. Asterisks denote the expected migration of each recombinant protein by SDS-PAGE. “Empty vector” and “MBP alone” represent eluate from *E*. *coli* cells harbouring pET30a or pMAL-c4e, respectively, with no insert. Molecular weight standards are shown.

**Fig 4:**
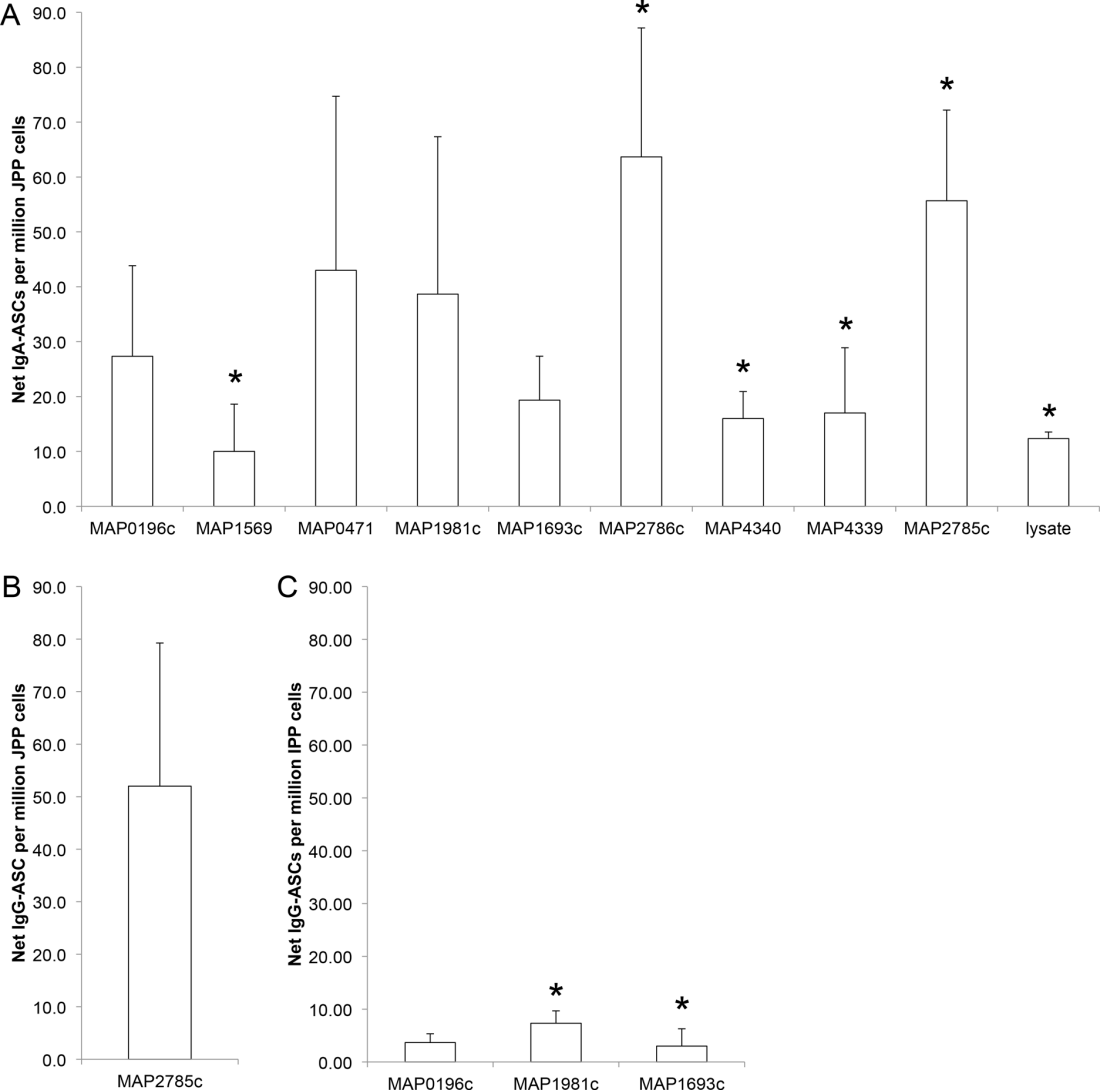
Net IgA-ASC and IgG-ASC responses to recombinant *M*. *avium* subsp. *paratuberculosis* proteins. Cell suspensions were prepared from PP collected from *M*. *avium* subsp. *paratuberculosis*-infected and uninfected compartments in mid-jejunal segments and assayed for IgA (A) and IgG (B) ASCs specific to recombinant *M*. *avium* subsp. *paratuberculosis* proteins. Cell suspensions were prepared from PP collected from *M*. *avium* subsp. *paratuberculosis*-infected and uninfected compartments in terminal small intestinal segments and assayed for IgG (C) ASCs specific to recombinant *M*. *avium* subsp. *paratuberculosis* proteins. The net number of ASC specific to each *M*. *avium* subsp. *paratuberculosis* protein in infected compartments was calculated using the formula provided in Materials and Methods. *M*. *avium* subsp. *paratuberculosis* proteins for which all calves (n = 3) had detectable net ASC responses are shown with significant (P < 0.05) increases in the frequency of *M*. *avium* subsp. *paratuberculosis*-protein specific ASCs denoted by an asterisk. Data presented are mean values with SEM.

IgA and IgG ASCs in *M*. *avium* subsp. *paratuberculosis*-infected and uninfected mid-jejunal and terminal small intestine compartments were enumerated by ELISPOT for each of the recombinant *M*. *avium* subsp. *paratuberculosis* proteins (Fig 3, Table 3). Net ASC responses were calculated as described in the Materials and Methods by subtracting the frequency of ASCs detected in the adjacent uninfected compartment from the *M*. *avium* subsp. *paratuberculosis*-infected compartment. Net IgA-ASC responses were detected for 9 of the 13 recombinant proteins (MAP0196c, MAP0471, MAP1569, MAP1693c, MAP1981c, MAP2785c, MAP2786c, MAP4340, MAP4339,) and *M*. *avium* subsp. *paratuberculosis* lysate when using PPs isolated from *M*. *avium* subsp. *paratuberculosis*-infected compartments in mid-jejunal segments from all three calves (Fig 4A). This Net-IgA response was significantly (P < 0.05) greater than in adjacent uninfected compartments for 5 of the 13 recombinant *M*. *avium* subsp. *paratuberculosis* proteins (MAP1569, MAP2785c, MAP2786c, MAP4339, MAP4340) and the *M*. *avium* subsp. *paratuberculosis* lysate (Table 3, Fig 4A). Although the magnitude of IgA-ASC frequency varied among the recombinant proteins tested the differences in frequencies were not statistically significant (P > 0.05) when analyzed with a one-way ANOVA. In contrast, no significant difference in IgA-ASCs frequency was observed with any of the 13 *M*. *avium* subsp. *paratuberculosis* recombinant proteins or bacterial lysate when analyzing PP cells isolated from *M*. *avium* subsp. *paratuberculosis*-infected and uninfected compartments in terminal small intestinal segments (data not shown). Moreover, the frequency of IgA-ASCs detected in the terminal small intestine segments was very low (< 10 ASC/million PP cells), and responses were never detected in all three calves (data not shown). The IgG-ASCs ELISPOT assay identified a greater, but not statistically significant, response to only MAP2785c recombinant protein with PP cells isolated from *M*. *avium* subsp. *paratuberculosis*-infected compartments in the mid-jejunum segment versus adjacent uninfected compartments (Fig 4B). A significant (P < 0.05) increase in IgG-ASCs specific to both the MAP1981c and MAP1693c proteins was also detected with cells from the *M*. *avium* subsp. *paratuberculosis*-infected IPP located in the terminal small intestine segment (Fig 4C). The frequency of these *M*. *avium* subsp. *paratuberculosis* protein-specific IgG plasma cells in the IPP was again very low (< 10 ASC/million PP cells) when compared to the frequency of IgG-ASCs specific for MAP2785c recombinant protein within the JPP (> 50 ASC/million PP cells). The method used to isolate PP cells from IPP and JPP involved disruption of the mucosal epithelium. Therefore, it is possible that these cell suspensions included cells derived from both submucosal lymphoid follicles (immune induction site) and the lamina propria (immune effector site) within intestinal villi.

**Table 3:** Summary of recombinant *M*. *avium* subsp. *paratuberculosis* proteins and bacterial lysate in which IgA and IgG ASC responses were detected in all three calves. The net frequency of IgA and IgG plasma cells was determined by comparing ASC frequency for individual proteins in *M*. *avium* subsp. *paratuberculosis*-infected and uninfected compartments of mid-jejunal segments (^1^) and in the terminal small intestine segments (^2^).

	Jejunal PP^1^		Ileal PP^2^	
ASC isotype	IgA	IgG	IgA	IgG
**Protein**				
**MAP0196c**	3			3
**MAP1569**	3*			
**MAP0471**	3			
**MAP1981c**	3			3*
**MAP1693c**	3			3*
**MAP2786c**	3*			
**MAP4340**	3*			
**MAP4339**	3*			
**MAP2785c**	3*	3		
**Lysate**	3*			
**MAP3634**				
**MAP3428c**				
**MAP1718c**				
**MAP0281**				

*Recombinant *M*. *avium* subsp. *paratuberculosis* proteins for which there was a significantly (P < 0.05) increased frequency of ASC in *M*. *avium* subsp. *paratuberculosis*-infected compartments than adjacent uninfected compartments.

### *M*. *avium* subsp. *paratuberculosis*-specific IgA in luminal contents of intestinal segments

**Fig 5:**
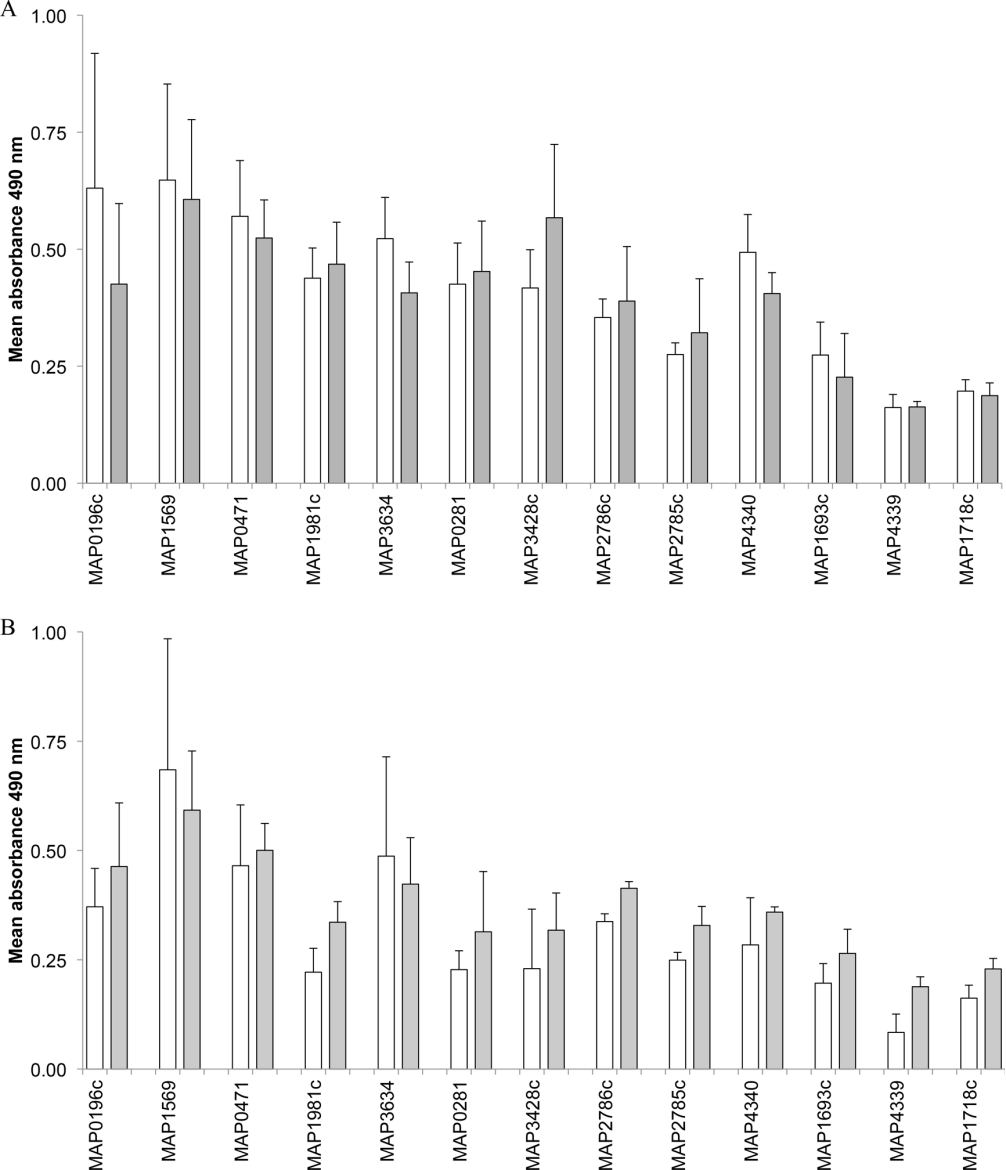
Secretory IgA in the intestinal contents of *M*. *avium* subsp. *paratuberculosis*-infected (grey bars) and uninfected (white bars) compartments collected from mid-jejunal (A) and terminal small intestinal (B) segments. Secretory IgA levels were measured using an IgA-specific ELISA with intestinal contents diluted 1:50. Intestinal samples were assayed from calves 6, 7, and 10, for which there was no evidence of *M*. *avium* subsp. *paratuberculosis* infection in the PBS injected compartments. Data presented are mean A_490_ values and error bars represent SEM.

Secretory IgA reacting with all 13 recombinant proteins was detected by ELISA when assaying intestinal contents collected from both *M*. *avium* subsp. *paratuberculosis*-infected and uninfected compartments in both the mid-jejunal and terminal small intestinal segments (Fig 5). The level of secretory IgA varied considerably, however, when comparing among *M*. *avium* subsp. *paratuberculosis* recombinant proteins and between mid-jejunal versus terminal small intestinal segments. For three proteins (MAP0281, MAP1981c, MAP3428c) the level of secretory IgA levels was consistently higher in mid-jejunal than terminal small intestinal segments but this difference was not significant. For the remaining 10 recombinant *M*. *avium* subsp. *paratuberculosis* proteins the level of secretory IgA was similar in both uninfected and *M*. *avium* subsp. *paratuberculosis*-infected compartments sampled from mid-jejunal and terminal small intestinal segments.

### Systemic IgG responses to recombinant proteins

**Fig 6:**
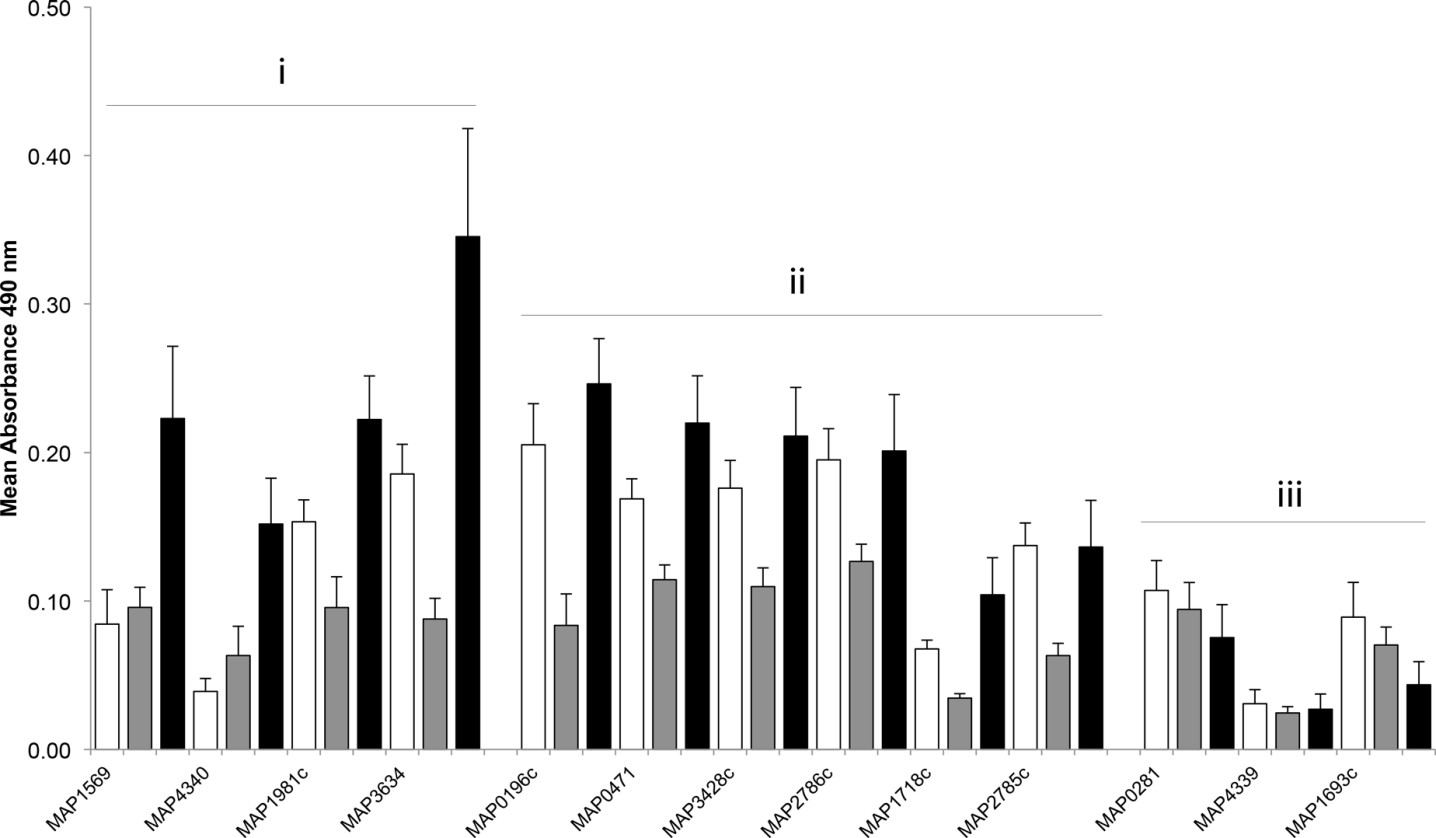
Serum IgG reacting with recombinant *M*. *avium* subsp. *paratuberculosis* proteins before and after injecting *M*. *avium* subsp. *paratuberculosis* into intestinal segments of 10 to 14 day old calves. Specific IgG levels were determined using an IgG-specific ELISA with sera diluted 1:600. For 10 of the 13 proteins, there was significantly (P < 0.05) increased reactivity with serum collected from calves at two month PI (black bars; n = 5) when compared to 1 month PI (grey bars; n = 5) (Groups i and ii). For 4 of these proteins (Group i), serum reactivity was significantly (P < 0.05) increased at 2 months PI when compared to both pre-infection (white bars) and one month PI. For three *M*. *avium* subsp. *paratuberculosis* proteins (Group iii) the level of serum reactivity did not change significantly following *M*. *avium* subsp. *paratuberculosis* infection. Data presented are mean A_490_ values with SEM.

Serum samples were collected immediately prior to surgery (n = 10) and when calves were euthanized at either one (n = 5) or two months PI (n = 5). The level of serum IgG reacting with individual *M*. *avium* subsp. *paratuberculosis* recombinant proteins was measured by ELISA. Serum IgG levels for 4 proteins (MAP1569, MAP1981c, MAP3634, MAP4340) were significantly (P < 0.05) greater at two months PI than either one month PI or pre-surgery (Fig 6). For 6 proteins (MAP0196c, MAP0471, MAP1718c, MAP2785c, MAP2786c, MAP3428c) serum IgG levels were significantly (P < 0.05) greater at two months PI than one month PI. Finally, for three of the recombinant proteins (MAP0281, MAP4339, and MAP1693C) no significant change in serum reactivity was observed following *M*. *avium* subsp. *paratuberculosis* injection into the intestinal segments.

### T-cell activation assays

The detection of isotype-switched ASC responses to multiple *M*. *avium* subsp. *paratuberculosis* proteins in JPP suggested the involvement of follicular T cells in the response to *M*. *avium* subsp. *paratuberculosis* infection. Therefore, cells isolated from PP located in *M*. *avium* subsp. *paratuberculosis*-infected and uninfected compartments of both mid-jejunal and terminal small intestinal segments were assayed for T cell responses. The analysis of T cell responses was limited to the three animals for which both IHC and PCR confirmed *M*. *avium* subsp. *paratuberculosis* was absent from the uninfected compartment in both mid-jejunal and terminal small intestinal segments (Tables 1 and 2). First, lymphocyte proliferative response (LPR) assays were performed using the 13 recombinant *M*. *avium* subsp. *paratuberculosis* proteins and *M*. *avium* subsp. *paratuberculosis* lysate. Significantly increased LPR responses were not detected, however, when comparing *M*. *avium* subsp. *paratuberculosis* protein-induced ^3^H-thymidine incorporation for PP cells isolated from *M*. *avium* subsp. *paratuberculosis*-infected versus uninfected compartments (data not shown). IFN-γsecreting cells (SC) were also enumerated using an ELISPOT assay following a 24 h *in vitro* re-stimulation of PP cells with each of the 13 recombinant *M*. *avium* subsp. *paratuberculosis* proteins and *M*. *avium* subsp. *paratuberculosis* lysate. Net IFN-γ secreting cells (SC) responses were calculated based on a comparison of PP cells isolated from *M*. *avium* subsp. *paratuberculosis*-infected and the adjacent uninfected compartment. Two recombinant proteins (MAP1569 and MAP1693c) and *M*. *avium* subsp. *paratuberculosis* lysate induced a greater frequency of IFN-γ SCs in PP cells isolated from *M*. *avium* subsp. *paratuberculosis*-infected mid-jejunal compartments but the increased frequency of IFN-γ SCs was significant (P < 0.05) for only recombinant MAP1693c protein (data not shown). In contrast, none of the *M*. *avium* subsp. *paratuberculosis* recombinant proteins or *M*. *avium* subsp. *paratuberculosis* lysate stimulated an increased IFN-γ SC response with cells isolated from the *M*. *avium* subsp. *paratuberculosis*-infected ileal PP from the terminal small intestinal segments (data not shown).

## Discussion

A variety of infection models have been used in young calves to target *M*. *avium* subsp. *paratuberculosis* infection to specific mucosal sites for investigation of host-pathogen interactions during the early phase of infection [9–11, 21]. One advantage of the current surgical model is that direct injection of *M*. *avium* subsp. *paratuberculosis* in the lumen preserved the natural route of bacterial invasion across the epithelial barrier and, as reported previously for ileal segments [12], *M*. *avium* subsp. *paratuberculosis* infection was localized to mid-jejunal and terminal small intestinal segments. IHC and PCR confirmed *M*. *avium* subsp. *paratuberculosis* uptake by both JPP and IPP (Figs 1 and 2; Tables 1 and 2). Localizing *M*. *avium* subsp. *paratuberculosis* invasion to single compartments within the two intestinal segments allowed us to directly compare the induction of pathogen-specific mucosal immune responses in two distinct types of PP and within the same animal thereby controlling for both genetic and inter-animal differences that potentially could result in distinct immune responses. One limitation of the model used in the current investigation is the potential for leakage of the infectious agent into adjacent control compartments. Specifically, we observed leakage of *M*. *avium* subsp. *paratuberculosis* from infected to adjacent control compartments in two of the 7 calves (Tables 1 and 2). The silk ligature used to divide compartments within each segment may not completely occlude the gut lumen throughout the entire infection period. Charavaryamath et al. (2013) separated *M*. *avium* subsp. *paratuberculosis*-infected and uninfected compartments in intestinal segments with an intervening compartment to prevent *M*. *avium* subsp. *paratuberculosis* infection of control compartments. Using a double ligature between infected and control compartments may be necessary to ensure localization of *M*. *avium* subsp. *paratuberculosis* infection to the injection site. To eliminate possible immune effects related to *M*. *avium* subsp. *paratuberculosis* leakage into control compartments, our analysis of mucosal immune responses in JPP and IPP was limited to three animals for which there was no evidence of *M*. *avium* subsp. *paratuberculosis* contamination within adjacent control compartments. This ensured the specificity of our analysis of IgA and IgG ASC responses but reduced the power of our analysis when comparing responses to individual recombinant proteins.

MLNs have been identified as a common site of *M*. *avium* subsp. *paratuberculosis* infection in naturally infected animals [6] and *M*. *avium* subsp. *paratuberculosis* can disseminate to draining lymph nodes within one hour after bacteria were injected into the lumen of an occluded segment of small intestine [10]. Therefore, it is not surprising that *M*. *avium* subsp. *paratuberculosis* was detected within the MLNs draining both mid-jejunal and terminal small intestinal segments of over half the calves in the current study (Tables 1 and 2). These results further confirm *M*. *avium* subsp. *paratuberculosis* uptake can occur throughout the small intestine. One limitation of the current infection model, however, was that it was not possible to determine whether lymphatic drainage from the *M*. *avium* subsp. *paratuberculosis*-infected and adjacent control compartments converged on the same MLN. Afferent lymphatic vessels originate within lacteals of intestinal villi and converge into an extensive lymphatic plexus within the deep submucosa of the intestine [22]. Without a negative control to assess the specificity of immune responses to recombinant *M*. *avium* subsp. *paratuberculosis* proteins it was not possible to determine if there were regional differences in the immune responses observed within mid-jejunal versus ileal MLNs. We did, however, detect IgA B cell responses in MLNs draining the *M*. *avium* subsp. *paratuberculosis*-infected mid-jejunal and terminal small intestine compartments for at least 6 of the recombinant proteins and *M*. *avium* subsp. *paratuberculosis* lysate (S3A and S3C Fig); IgG B cell responses were evident with a smaller subset of proteins and showed a lower magnitude of response (S3B and S3D Fig). These immune responses further support the observation that *M*. *avium* subsp. *paratuberculosis* migrated beyond the PP into the MLN. Moreover, MLN are critical amplification sites for effector immune cells, such as IgA plasma cells, where imprinting of mucosal homing receptors occurs which is necessary for trafficking of these cells back to the mucosal surface of the small intestine. The induction of IgA and IgG plasma cells in MLN may have contributed to the detection of secretory IgA in all intestinal segments and may explain the detection of IgG ASCs in cell suspensions prepared from the IPP.

In naturally infected cattle and small ruminants, lesions associated with Johne’s disease are most prevalent in the terminal small intestine but frequently involve MLN throughout the small intestine [6, 23]. The biased distribution of lesions in the small intestine may have influenced earlier investigations which confirmed M cells in the IPP provide an efficient portal of entry for *M*. *avium* subsp. *paratuberculosis* [3, 4]. Recently, however, it was reported that *M*. *avium* subsp. *paratuberculosis* was taken up with equal efficiency by mucosal epithelium and M cells in the follicle-associated epithelium of ovine JPP and IPP [13]. Our study confirms that *M*. *avium* subsp. *paratuberculosis* can be taken up by JPP and IPP in newborn calves, resulting in the accumulation of bacterial protein within submucosal lymphoid follicles and associated dome regions (Figs 1 and 2). The accumulation of *M*. *avium* subsp. *paratuberculosis* antigen within lymphoid follicles of the PP is consistent with its entry through M cells in the follicle associated epithelium. Moreover, the high frequency of lymphoid follicles stained by IHC in both JPP and IPP suggests that *M*. *avium* subsp. *paratuberculosis* is sampled efficiently by both PP in young calves. This observation may have further significance when considering that the primary route of *M*. *avium* subsp. *paratuberculosis* infection in young calves is through fecal-oral transmission. The jejunum is approximately 10 times greater in length than the ileum and containing 25–40 discrete PPs [24]. Therefore, it is probable that tonsils and the JPP may be the first mucosa-associated lymphoid tissues to sample *M*. *avium* subsp. *paratuberculosis*.

Marked differences were observed when comparing pathogen-specific IgA antibody responses induced in JPP versus IPP (Fig 4; Table 3). IgA-ASCs responses were detectable with 9 of the 13 recombinant *M*. *avium* subsp. *paratuberculosis* proteins when comparing infected versus uninfected JPP in all three calves tested. In contrast, no IgA-ASC response was detected with any of the recombinant *M*. *avium* subsp. *paratuberculosis* proteins when comparing infected versus uninfected IPP. This remarkable difference in IgA B cell responses is consistent, however, with a previous report that IgA-ASC responses were not detected following selective targeting of a viral vaccine vector to IPP in the small intestine of newborn sheep [15]. The generation of IgA plasma cells in mucosa-associated lymphoid tissues is dependent on cognate T cell-B cell interactions that result in isotype-switching [25]. Thus, a failure to detect IgA-ASC responses specific to *M*. *avium* subsp. *paratuberculosis* proteins in the IPP is consistent with previous reports that follicular CD4^+^ T cells constitute less than 0.5% of the cells present in lymphoid follicles of the IPP [14]. This is in contrast to the abundance of follicular CD4^+^ T cells (10–15%) in lymphoid follicles of JPP [14]. A recent analysis of B cell activation and somatic mutation in bovine IPP supports the conclusion that this PP functions as a site for antigen-independent diversification of the pre-immune B cell repertoire [26]. Thus, our data (Fig 4, Table 3) provides the first evidence that *M*. *avium* subsp. *paratuberculosis* uptake by M cells in the IPP may provide this pathogen with a portal of entry that effectively evades the induction of a local mucosal IgA antibody response.

Further, IPP follicles contain an abundance of tangible-body (TB) macrophages to remove the apoptotic bodies arising from the extensive programmed cell death of immature B cells in IPP [27]. Ingestion of apoptotic cells down-regulates macrophage pro-inflammatory activity [28] and it remains to be determined whether TB macrophages have a reduced capacity to clear *M*. *avium* subsp. *paratuberculosis* infection. The absence of antigen-dependent B cell activation, combined with reduced macrophage function would make IPP an ideal location for *M*. *avium* subsp. *paratuberculosis* to establish a persistent infection. The induction of specific mucosal immune responses at other inductive sites, such as JPP or MLN, may be able to block further uptake of *M*. *avium* subsp. *paratuberculosis* from the intestinal lumen. The homing of mucosal effector cells to the lamina propria would, however, have no effect on bacterial infections already established within lymphoid follicles of the IPP. Further investigation may also be warranted to determine whether the presence of foreign bacterial proteins within lymphoid follicles of the IPP tissue alters selection of the B cell pre-immune repertoire.

Secretory IgA reacting with the 13 recombinant *M*. *avium* subsp. *paratuberculosis* proteins was detected in luminal contents collected from both *M*. *avium* subsp. *paratuberculosis*-infected and uninfected compartments in both mid-jejunal and terminal small intestinal segments (Fig 5). A similar level of secretory IgA was detected among all intestinal compartments for 10 of the 13 recombinant proteins but a higher level of secretory IgA was detected in jejunal versus ileal compartments with three recombinant proteins. It is interesting to note that there was no direct correlation between the magnitude of IgA-ASC responses to individual *M*. *avium* subsp. *paratuberculosis* proteins in JPP (Fig 4) and the level of secretory IgA detected in intestinal contents (Fig 5). These results may be explained by the homing of IgA B cells from multiple immune induction sites, such as the JPP (Fig 4) and draining MLN (S3 Fig) to mucosal epithelium throughout the small intestine [29]. Our current observations regarding the presence of *M*. *avium* subsp. *paratuberculosis* protein-specific IgA in the lumen of both jejunum and ileum (Fig 5) may have significant implications for oral vaccine design and delivery. *M*. *avium* subsp. *paratuberculosis* uptake by JPP and the draining MLN has the potential to generate IgA plasma cells that traffic throughout the small intestine. If these IgA plasma cells produce secretory IgA targeting attachment or invasion proteins then it may be possible to block *M*. *avium* subsp. *paratuberculosis* invasion of the IPP. Fecal IgA responses were recently identified as a possible correlate of protection in sheep infected with *M*. *avium* subsp. *paratuberculosis* [30]. *In vitro* studies have also demonstrated that antibody opsonization of *M*. *avium* subsp. *paratuberculosis* may increase bacterial killing through Fc-receptor mediated uptake by activated bovine macrophages [31, 32]. Further studies are needed, however, to test *in vivo* that secretory IgA responses can prevent *M*. *avium* subsp. *paratuberculosis* invasion of the ileum. One potential vaccine candidate may be MAP1569, which contains a fibronectin-attachment domain that mediates *M*. *avium* subsp. *paratuberculosis* adhesion to M cells [33] and which the current study confirmed was highly immunogenic (Table 3). Finally, the presence of secretory IgA specific for multiple *M*. *avium* subsp. *paratuberculosis* proteins in the intestinal contents early after infection (Fig 5) suggests that testing fecal IgA may provide a novel strategy for early detection of *M*. *avium* subsp. *paratuberculosis* infection.

The analysis of serum following enteric infection of newborn calves identified 10 *M*. *avium* subsp. *paratuberculosis* proteins for which there were increased IgG antibody levels at 2 months PI (Fig 6). For 7 of these proteins a corresponding induction of an IgA-ASC response was detected in the JPP. Furthermore, for three of the 4 proteins for which IgG-ASC responses were detected in PPs there were also increased serum IgG antibody levels at 2 months PI. Interestingly, IgG reactivity was observed with 6 of the recombinant *M*. *avium* subsp. *paratuberculosis* proteins with serum collected from newborn calves prior to infection. The marked decline in serum IgG reactivity with these proteins one month later suggests that this reactivity was due to maternal antibody (Fig 6). This is somewhat surprising since the dams of all calves tested seronegative with the commercial IDEXX MAP Ab Test. Serum IgG reactivity with recombinant *M*. *avium* subsp. *paratuberculosis* proteins in newborn calves may also reflect the transfer of cross-reactive maternal antibodies that were induced by prior exposure to other environmental mycobacterial species. Other groups have reported serum antibody responses first detected at 70 days to 4.4 months after calves were either orally fed or had *M*. *avium* subsp. *paratuberculosis* administered by tonsillar instillation [8, 34, 35]. This route of bacterial challenge has been postulated to simulate the natural route of infection and stimulate earlier humoral responses than more artificial intestinal infection models [10]. Two possible differences in our study design that may account for earlier detection of serum antibody responses may be targeting infection to JPP and using individual *M*. *avium* subsp. *paratuberculosis* proteins for our ELISA. Western blot analysis had previously revealed antibodies reacting with two unidentified *M*. *avium* subsp. *paratuberculosis* proteins were present in serum within two weeks after tonsillar instillation [8]. Thus, our results may more closely reflect tonsillar instillation where *M*. *avium* subsp. *paratuberculosis* is being delivered directly to a known mucosal immune induction site.

We also compared the induction of *M*. *avium* subsp. *paratuberculosis*-specific T cell responses in JPP and IPP. We anticipated that T cell responses specific to the recombinant *M*. *avium* subsp. *paratuberculosis* proteins would occur in concert with IgA-ASC responses in the JPP, however none of the recombinant *M*. *avium* subsp. *paratuberculosis* proteins induced significant LPR with cells isolated from either the JPP or IPP. Two recombinant proteins, MAP1569 and MAP1693c, as well as *M*. *avium* subsp. *paratuberculosis* lysate induced significant IFN-γ SC responses with cells isolated from JPP, but no response was evident with IPP cells. In cells isolated from MLNs draining the terminal small intestine 5 of the 13 recombinant proteins induced weak IFN-γ SC responses (< 10 ASCs per million), but only *M*. *avium* subsp. *paratuberculosis* lysate induced IFN-γSC response in cells isolated from both jejunal and ileal MLNs draining *M*. *avium* subsp. *paratuberculosis*-infected segments (S4 Fig). Other studies have reported cell-mediated responses in *M*. *avium* subsp. *paratuberculosis*-infected calves when re-stimulating blood leukocytes or MLN cells i*n vitro* with *M*. *avium* subsp. *paratuberculosis* lysate, however these responses were measured much later in infection at 9–11 months [10, 12]. The natural progression of Johne’s disease suggests a dissemination of *M*. *avium* subsp. *paratuberculosis* from the submucosa to the draining MLN subsequently leading to lesions developing in lymphoid tissue [6]. These results may indicate that mucosal immune induction sites, such as JPP, may not be the optimal tissue for the detection of T cell responses early after *M*. *avium* subsp. *paratuberculosis* infection (< 3 months). The extensive distribution of CD4^+^ and CD8^+^ T cells within the lamina propria and intraepithelial leukocyte compartments, respectively, of newborn calves [19] may make these immune compartments more appropriate sites for monitoring the early induction of antigen-specific effector T cells.

In conclusion, we developed an animal model to target *M*. *avium* subsp. *paratuberculosis* infection to specific gut-associated lymphoid tissues and investigated whether the portal of *M*. *avium* subsp. *paratuberculosis* infection significantly altered induction of mucosal immune responses. Using 13 recombinant *M*. *avium* subsp. *paratuberculosis* proteins we clearly demonstrated the functional dichotomy between JPP and IPP following *M*. *avium* subsp. *paratuberculosis* uptake into lymphoid follicles. *M*. *avium* subsp. *paratuberculosis* infection of JPP, but not IPP, induced IgA-ASC responses detectable with 9 of the 13 recombinant *M*. *avium* subsp. *paratuberculosis* proteins and bacterial lysate. Despite localized induction of *M*. *avium* subsp. *paratuberculosis*-specific antibody responses in the JPP, it was possible to detect secretory IgA reacting with recombinant *M*. *avium* subsp. *paratuberculosis* proteins in luminal contents collected from both jejunal and ileal intestinal segments. These observations have broader implications for the pathobiology of Johne’s disease and the possible development of diagnostic tools as well as vaccine targets. The failure to induce mucosal immune responses within IPP suggests immune evasion strategies by *M*. *avium* subsp. *paratuberculosis* to establish persistent infections. Other enteric pathogens may employ similar mechanisms. The current observation, however, that mucosal immune responses to multiple *M*. *avium* subsp. *paratuberculosis* proteins were induced in JPP within one to two months PI indicates it may be possible to vaccinate calves early in life and induce immune responses at a time when animals are most vulnerable to *M*. *avium* subsp. *paratuberculosis* infection [2]. These observations provide the foundation for validating the immunogenicity and potential utility of individual *M*. *avium* subsp. *paratuberculosis* proteins for either diagnostic tests or vaccine candidates.

## Materials and Methods

### Ethics Statement

All animal experiments were conducted at the University of Saskatchewan in accordance with regulations approved by the Canadian Council on Animal Care and endorsed by the University of Saskatchewan Animal Care Committee (Protocol 20020105).

### Animals, surgery, and *M*. *avium* subsp. *paratuberculosis*-infection

Calves were obtained from dams that tested seronegative for *M*. *avium* subsp. *paratuberculosis* by the IDEXX MAP Ab Test (IDEXX Laboratories, Westbrook, ME). Procedures for housing and feeding calves, anesthesia, surgery, and post-surgical care were previously described [36]. Briefly, 10 to 14 day-old, male Holstein calves (n = 7) received 3 L colostrum within 3–6 h postpartum and were then fed 3 L pasteurized milk twice daily. Calves were fasted for 12 h prior to surgery and one mL diazepam (5 mg/mL; Sandoz Canada Inc., Boucherville, QC) and one mL butorphanol tartrate (0.2 mg/kg; Torbugesic, Wyeth Animal Health, Guelph, ON) were injected intravenously. Anaesthesia was induced with an intravenous injection of thiopental sodium (6–10 mg/kg, Pentothal, Hospira Healthcare Corp., Vaughan, ON), an endotracheal tube was inserted and anaesthesia was maintained with 2–3% Isoflurane (AErrane, Baxter, Mississauga, ON) in 100% oxygen and intermittent positive pressure ventilation. The small intestine, immediately anterior to the ileocecal fold, was exteriorized through a paralumbar abdominal incision and a 20-cm segment of intestine was demarcated at each end with 2 consecutive intestinal clamps. Intestine was transected between each pair of clamps and ingesta in the isolated segment of intestine was removed by flushing with magnesium- and calcium-free phosphate buffered saline (PBSA). The lumen of the segment was then infused with 250 mg metronidazole (Hospira Healthcare Corp., Montreal, QC, Canada) and 200 mg enrofloxacin (Baytril; Bayer Incorp., Toronto, ON, Canada) for 25 min. Continuity of the gastrointestinal tract was re-established by re-aligning mesenteric and anti-mesenteric borders of the intestine proximal and distal to the isolated segment and performing an end-to-end anastomosis. Antibiotics were removed from the isolated segment by flushing with PBSA, and the intestinal segment was then oversewn at both ends. The isolated segment was subdivided into two compartments using a silk ligature to occlude the intestinal lumen, and each compartment was injected with 1 g neomycin (Neomix; Pharmacia & Upjohn Animal Health, Orangeville, ON, Canada), 1.2 g florfenicol (Nuflor; Merck Canada Inc., Kirkland, QC, Canada), and 250 mg metronidazole in 5 ml PBSA. The distal compartment was then injected with 10^9^ CFU of bovine *M*. *avium* subsp. *paratuberculosis* isolate gc86 [20] re-suspended in 5 mL PBSA. The proximal compartment of the segment was injected with 5 mL PBSA. A second intestinal segment was then prepared in the mid-jejunal region following a procedure similar to that described for the preparation of the terminal small intestinal segment. Briefly, two consecutive JPP were identified by examining the serosal surface of the jejunum and a variable length of jejunum, encompassing the two discrete PPs, was demarcated by placing two consecutive intestinal clamps at the proximal and distal end of the segment. The isolated jejunal segment was flushed and treated with antibiotics as described for the previous segment before using silk ligatures to subdivide the jejunal segment into 3 compartments. The distal compartment, containing a discrete PP, was inoculated with 10^9^ CFU *M*. *avium* subsp. *paratuberculosis* isolate gc86 re-suspended in 5 mL PBSA. The proximal compartment, also containing a discrete PP, was injected with 5 mL PBS. The two intestinal segments were returned to the abdominal cavity and the incision enclosed as described previously [12]. Post-surgically, all animals were treated daily with 1.1 mg/kg flunixin (Banamine; Schering Plough Canada Inc., Pointe Claire, PQ, Canada) and 3–4 mg/kg enrofloxacine (Baytril; Bayer Inc.) to minimize pain for up to five days post-surgery.

A preliminary experiment was performed with three calves to establish and optimize immune assays with recombinant *M*. *avium* subsp. *paratuberculosis* proteins. These calves were not included in the analysis of mucosal immune responses as *M*. *avium* subsp. *paratuberculosis* was injected into mid-jejunal and terminal small intestinal segments consisting of a single compartment. Serum samples collected when these calves were euthanized at one month PI were, however, included in the analysis of serum IgG responses.

### Pathology, histology, and PCR detection of *M*. *avium* subsp. *paratuberculosis*

Calves were euthanized by intravenous injection of Euthanyl (20 mL/45 kg body weight; Bimeda-MTC, Canada) at one or two months PI and tissues were collected within 10–15 minutes. Surgically isolated segments, adjacent intestine, and MLN were examined and photographed to record gross anatomy. Tissue samples were fixed in 10% neutral-buffered formalin for histological examination. Mid-jejunal and terminal small intestinal segments were opened along the mesenteric border, intestinal contents collected, and the mucosal surface examined and photographed to record gross anatomy. Intestinal contents were collected in sterile tubes containing 10 mL PBS, gently mixed, and centrifuged at 3,000 x g for 5 min to collect clarified supernatant for quantification of *M*. *avium* subsp. *paratuberculosis*-specific secretory (s)IgA. Tissue embedding, sectioning, hematoxylin and eosin (H&E), and immunohistochemical staining for *M*. *avium* subsp. *paratuberculosis* was performed by Prairie Diagnostic Services (Saskatoon, SK, Canada).

PCR amplification was used to detect *M*. *avium* subsp. *paratuberculosis* in paraffin-embedded tissue sections from mid-jejunal uninfected (n = 7) and infected (n = 7) compartments and terminal small intestine uninfected (n = 7) and infected (n = 7) compartments and adjacent draining MLNs. DNA extraction and PCR were performed by Prairie Diagnostic Services. Briefly, DNA from paraffin-embedded tissue was extracted using the DNeasy Blood and Tissue Kit (Qiagen Inc.) and following the manufacturer’s instructions. PCR was performed using the VetAlert™ Johne’s Real-Time PCR kit specific for *M*. *avium* subsp. *paratuberculosis* (Tetracore Inc., Rockville, MD).

### Preparation of *M*. *avium* subsp. *paratuberculosis* secreted proteins

*M*. *avium* subsp. *paratuberculosis* strain gc86 cultured in Middlebrook 7H9 medium was used to prepare whole-cell lysate. Bacteria were pelleted, and the pellet re-suspended at a final concentration of 1.34 x 10^8^ CFU/mL in PBSA and sonicated with a high-intensity ultrasonic processor (Vibra-Cell, Danbury, CT) at 40% amplitude with alternate cycles of 8 s on and 8 s off for 10 min. The resulting cell lysate was centrifuged at 12,000 x *g* for 5 min, the pellet discarded, and protein concentration of the soluble fraction was measured using a Quant-iT protein assay kit (Invitrogen). Phenylmethylsulfonyl fluoride (PMSF) was added to the lysate at a final concentration of 1 mM before aliquots were stored at –20°C.

A summary of all recombinant proteins evaluated in this study is provided in S1 Table. Genes encoding *M*. *avium* subsp. *paratuberculosis* secreted proteins were cloned in a pET-30a or pMal-c4e vector. *E*.*coli* recombinant pET-30a clones were cultured in LB broth supplemented with 50 μg/mL kanamycin and 35 μg/mL chloramphenicol to an OD_600_ of 0.6 at 37°C. Protein expression was induced with 0.1 mM IPTG and cultured for an additional 16 h at 25°C. Cells were pelleted, suspended in lysis buffer [50 mM NaH_2_PO_4_, 300 mM NaCl, 20 mM imidazole, 1 mg/mL lysozyme, 1 mM PMSF, pH 8.0], lysed by sonication, and soluble protein separated from the cellular debris by centrifugation at 8,500 x g for 15 min. Recombinant protein containing a carboxy-terminal 6xHistidine tag was captured from the soluble protein extract by nickel-NTA chromatography (Clontech Laboratories, Inc.), washed extensively with native-purification buffer [NPB; 50 mM Na_2_HPO_4_, 300 mM NaCl, pH 8.0] containing 90 mM imidazole and 0.1% v/v Triton X-114, and bound protein eluted with NPB containing 400 mM imidazole.

*E*.*coli* recombinant pMal-c4e clones were cultured in LB supplemented with 100 μg/mL ampicillin and 35 μg/mL chloramphenicol to an OD_600_ of 0.6 at 37°C. Protein expression was induced with 0.1 mM IPTG and bacteria were cultured for an additional 5 h at 37°C. Cells were harvested by centrifugation, suspended in column buffer [CB; 20 mM Tris-HCl, 50 mM NaCl, 1 mM EDTA, 1 mg/mL lysozyme, 1 mM PMSF, pH 7.5], lysed by sonication, and soluble protein separated from cellular debris by centrifugation. Recombinant protein fused to the carboxy-terminus of maltose-binding protein was captured from the soluble protein extract by amylose resin chromatography (New England Biolabs, Inc.). Soluble protein extract was diluted 1:5 in CB, incubated with the resin with gentle agitation for 1 h at 4°C, packed into a column, washed extensively with CB, and bound protein eluted with CB containing 10 mM maltose.

Eluted protein fractions were pooled, concentrated, and dialyzed against 10 mM PBS, pH 7.2 using Amicon Ultra-15 centrifugal filter units with a 10 kDa nominal molecular weight limit (NMWL; EMD Millipore). Protein concentration was quantified using a bicinchoninic acid kit (Sigma-Aldrich), and qualitatively analyzed by SDS-PAGE (Fig 3). *E*. *coli* cells harboring the pET30a or pMAL-c4e plasmid with no insert were subjected to the same culture conditions and chromatography described above. These elution fractions were pooled, dialyzed, and used in immune assays as “control eluate protein” to account for non-specific reactivity arising from *E*. *coli* maltose-binding protein, chromatography impurities, endogenous antigen, and/or endotoxin.

### Leukocyte isolation procedures

MLNs draining each intestinal segment were identified and collected based on their proximal location in the attached mesentery. Pericapsular fat and connective tissue was removed from each lymph node before cutting 1-cm^3^ pieces and immersing them in sterile PBSA. Each tissue piece was finely minced to release cells and the cell suspension was passed through a 40 μm nylon cell strainer. Cell suspensions were re-suspended in AIM V medium (Gibco) supplemented with 10% fetal bovine serum (FBS) plus antibiotics and antimycotics at a final concentration of 2 x 10^6^ cells/mL for lymphocyte proliferative response (LPR) assays or 5 x 10^6^ cells/mL for ELISPOT assays.

Non-PP intestinal tissue was removed from the margins of each PP before immersing tissue in AIM V medium supplemented with 10% FBS plus antibiotics and antimycotics. The edge of a scalpel blade was gently scraped over the mucosal surface of each PP to remove ingesta and mucous before transferring the PP to fresh medium. An incision was then made through the mucosal surface down to the serosal/muscularis layer and the scalpel blade was held at a 60° angle while scraping along the muscularis to elevate the mucosal layer and release submucosal lymphoid follicles into the medium. Sheets of mucosal epithelia were removed using forceps, and a single cell suspension prepared from lymphoid follicles by repeated pipetting with a 10 mL serological pipette. The cell suspension was passed through a 40 μm nylon cell strainer, pelleted, and then re-suspended in fresh AIM V medium supplemented with 10% FBS plus antibiotics and antimycotics at a final concentration of 2 x 10^6^ cells/mL for LPR assays or 5 x 10^6^ cells/mL for ELISPOT assays.

### Immune assays

#### ELISPOT assay for antibody-secreting cells (ASCs)

Recombinant *M*. *avium* subsp. *paratuberculosis* proteins (2 μg/mL), *M*. *avium* subsp. *paratuberculosis* lysate (5 μg/mL), and control eluate protein (2 μg/mL) were diluted in coating buffer (50 mM carbonate-bicarbonate, pH 9.6) before adding 100 μL/well to duplicate wells in a 96-well Multiscreen-HA plates (EMD Millipore) and incubating overnight at 4°C. After washing with coating buffer, wells were blocked with 200 μL/well Dulbecco’s Modified Eagle Medium (DMEM; Gibco) supplemented with 5% FBS for 2 h at 37°C. Cell suspensions from PPs and MLN (5 x 10^5^/well) were added to duplicate wells for each protein assayed and incubated in a humidified chamber at 37°C under 5% CO_2_ for 18 hours. Non-adherent cells were removed from the well and adherent cells lysed by washing twice with sterile water for 5 min. Wells were then washed three times with Tris-buffered saline (TBS) containing 0.05% Tween-20. Primary (either biotin conjugated anti-bovine IgG or IgA; AbD Serotec Ltd.) and secondary (alkaline-phosphatase conjugated streptavidin; Bio-Rad Laboratories Ltd.) antibodies were diluted 1:1,000 in TBS-T. All incubations were performed at 37°C for 1 h. Wells were then washed 5 times with TBS-T and 5 times with TBS between each antibody incubation. After extensive washing of wells with sterile water, 100 μL of BCIP/NBT reagent (Life Technologies Inc.) was added to each well for 5 min and the reaction stopped by washing with water. Well images were captured using the AID Elispot Reader ELR07 instrument, and spots enumerated using the AID Elispot Reader Software V7.0 (Autoimmun Diagnostika GmbH, Strassberg, Germany). The number of ASCs per million PP cells for each recombinant *M*. *avium* subsp. *paratuberculosis* protein was calculated using the formula: [(# of ASCs for recombinant protein–# of ASCs for medium alone)–(# of ASCs for control eluate protein–# of ASCs for medium alone)] x 2. The “Net Ig-ASCs” for each recombinant *M*. *avium* subsp. *paratuberculosis* protein of each antibody isotype was calculated as follows: (number of ASCs per million PP cells in the *M*. *avium* subsp. *paratuberculosis*-infected compartment)–(number of ASCs per million PP cells in the adjacent uninfected compartment).

#### ELISPOT assay for IFN-γ secreting cells

The mouse monoclonal anti-IFN-γ capture antibody was diluted 1:3,000 in coating buffer before adding 100 μL/well to 96-well Multiscreen-HA plates and incubating plates overnight at 4°C. After washing with coating buffer, wells were blocked with 200 μL DMEM supplemented with 5% FBS for 2 h at 37°C. Cell suspensions from PPs and MLNs (5 x 10^5^/well) were cultured in duplicate wells and stimulated with either medium alone, *M*. *avium* subsp. *paratuberculosis* lysate (1.5 μg/mL), recombinant *M*. *avium* subsp. *paratuberculosis* protein (1.5 μg/mL), or control eluate protein (1.5 μg/mL) and incubated in a humidified chamber at 37°C under 5% CO_2_ for 18 hours. The plates were processed as described above with the following changes: IFN-γ captured by the monoclonal antibody was detected with rabbit anti-bovine IFN-γ (1:1,500) followed by detection of rabbit antibody with HRP conjugated goat anti-rabbit IgG (1:1,000). Immunocomplexes were visualized using PBS containing 0.2% w/v diaminobenzidine and 0.03% H_2_O_2_. Well images were captured using the AID Elispot Reader ELR07 instrument, and spots enumerated using the AID Elispot Reader Software V7.0 (Autoimmun Diagnostika GmbH, Strassberg, Germany). The number of IFN-γ SCs per million PP cells for each recombinant *M*. *avium* subsp. *paratuberculosis* protein was calculated using the formula: ([number of IFN-γ SCs for recombinant protein–number of IFN-γ SCs for medium alone]–[number of IFN-γ SCs for control eluate protein–number of IFN-γ SCs for medium alone]) x 2. The “Net IFN-γ SCs” for each recombinant protein was calculated as follows: (number of IFN-γ SCs per million PP cells in *M*. *avium* subsp. *paratuberculosis*-infected compartment)–(number of IFN-γ SCs per million PP cells in the adjacent control compartment).

#### Lymphocyte proliferative response (LPR) assay

PP and MLN cells were plated at 2 x 10^5^ cells/well in a 96-well tissue culture plates in a final volume of 200 μL/well of AIM V medium supplemented with 10% FBS plus antibiotics and antimycotics. Triplicate cultures were stimulated with either medium alone, 0.025 μg/mL concanavalian A, 0.5 μg/mL *M*. *avium* subsp. *paratuberculosis* lysate, 0.5 μg/mL recombinant *M*. *avium* subsp. *paratuberculosis* protein, or 0.5 μg/mL control eluate protein. All cell stimuli were prepared in AIM V supplemented with 10% FBS and 10 ng/mL recombinant human IL-2. Cells were incubated in a humidified chamber at 37°C under 5% CO_2_ for 4 days, and during the last 24 h of culture 0.4 μCi [^3^H] thymidine was added to each well. Cells were lysed with two freeze-thaw cycles before harvesting DNA with a microplate cell harvester. Incorporation of [^3^H] thymidine was measured using a liquid scintillation counter (Beckman 1701). T cell proliferative responses were expressed as a stimulation index (SI), where SI = [cpm for cell suspensions stimulated with protein–cpm for cell suspensions in medium alone] / [cpm for cell suspensions stimulated with control eluate protein–cpm for cell suspensions in medium alone].

### ELISA detection of serum IgG and secretory IgA

Recombinant *M*. *avium* subsp. *paratuberculosis* proteins were diluted to 2 μg/mL in 50 mM carbonate-bicarbonate buffer pH 9.2, and 100 μL/well was added to Nunc Maxisorp 96-well flat bottom plates and incubated overnight at 4°C. After washing 3 times with PBS containing 0.05% Tween-20 (PBS-T), wells were blocked with 200 μL PBS-T containing 3% BSA for 2 h at 37°C. Bovine serum (1:300) or clarified intestinal contents (1:50) diluted in PBS-T containing 1% BSA was added to duplicate wells for each protein and incubated for 2 h at 37°C. Wells were washed 6 times with PBS-T after incubation with each of the following antibodies. For IgG quantification, HRP-conjugated rabbit anti-bovine IgG was diluted 1:3,000 in PBS-T-1% BSA and 100 μL was added to each well and incubated for 2 h at 37°C. For IgA quantification, biotin-conjugated rabbit anti-bovine IgA was diluted 1:5,000 in PBS-T-1% BSA and reacted for 1.5 h at 37°C, followed by HRP-conjugated streptavidin (1:10,000 in PBS-T-1% BSA) for 1 h at 37°C. Each well was reacted with 100 μL of o-phenylenediamine dihydrochloride (Sigma-Aldrich) diluted in phosphate-citrate buffer, pH 5.0 containing 0.03% H_2_O_2_ for 5 min. Colorimetric reactions were stopped by adding 50 μL of 2 N H_2_SO_4_, and absorbance read at 490 nm (Molecular Devices Spectra Max 340PC).

### Statistical analysis

A paired Student’s t-test was used to determine significance between ASCs, IFN-γ SCs, and LPRs between *M*. *avium* subsp. *paratuberculosis* infected and uninfected compartments in mid-jejunal and terminal small intestinal compartments. A one-way ANOVA was conducted to determine significant differences in the frequency of ASCs among *M*. *avium* subsp. *paratuberculosis* recombinant proteins and lysate. Serum IgG responses were grouped as pre-*M*. *avium* subsp. *paratuberculosis* infection (n = 10), one month PI (n = 5), or two months PI (n = 5), and compared using an unpaired Student’s t-test. Statistical significance is reported when P ≤ 0.05, mean values are presented for all data, and error bars represent the standard error of the mean.

## Supporting Information

S1 Fig Gross anatomy and histology of surgically isolated intestinal segments. Representative gross anatomy of mid-jejunal (A) and terminal small intestine (B) segment consisting of 2 or 3 compartments: C, control compartment injected with PBS; I, interspace compartment; M, *M*. *avium* subsp. *paratuberculosis*-infected compartment inoculated with 10^9^ CFUs. Arrow denotes the site of anastomosis to re-establish continuity of the intestinal tract. Representative picture of the gross appearance of the mucosal surface of discrete JPP (C) and continuous IPP (D) with PP demarcated by black lines. Hematoxylin and eosin staining of JPP (E) and IPP (F) from *M*. *avium* subsp. *paratuberculosis-*infected compartments. Ca, capsule; D, dome region; FAE, follicle associated epithelium; Fol, follicle; IF, interfollicular region; LN, lymph node; Lys, lymphatic sinus; Mus muc, muscularis mucosa; V, villus.
(TIF)

S2 Fig Histology and immunohistochemistry of an uninfected compartment 2 months post-surgical isolation and located adjacent to a *M*. *avium* subsp. *paratuberculosis*-infected compartment. Hematoxylin and eosin stain of IPP (a) and JPP (b). Immunohistochemistry of IPP (c) and JPP (d) with rabbit anti-*M*. *avium* subsp. *paratuberculosis* antibodies.
(TIF)

S3 Fig **Frequency of ASCs specific for *M*. *avium* subsp. *paratuberculosis* recombinant proteins and lysate in cell suspensions prepared from MLNs draining *M*. *avium* subsp. *paratuberculosis*-infected mid-jejunal (A, B) and terminal small intestinal (C, D) segments.** Mean values of IgA- (A, C) and IgG- (B, D) ASCs are presented with SEM.
(TIF)

S4 Fig Frequency of IFN-gamma secreting cells in response to recombinant *M*. *avium* subsp. *paratuberculosis* proteins and lysate in cell suspensions prepared from MLNs draining *M*. *avium* subsp. *paratuberculosis*-infected mid-jejunal (white bar) and terminal small intestine (grey bars) segments. Data presented are mean values with SEM.
(TIF)

S1 Table Recombinant *M*. *avium* subsp. *paratuberculosis* proteins used to evaluate immune responses. (PDF)

## References

[1] Harris NB, Barletta RG. *Mycobacterium avium* subsp. *paratuberculosis* in veterinary medicine. Clin Microbiol Rev 2001;14: 489–512. 11432810 10.1128/CMR.14.3.489-512.2001 PMC88986

[2] Benedictus A, Mitchell RM, Linde-Widmann M, Sweeney R, Fyock T, Schukken YH, et al Transmission parameters of *Mycobacterium avium* subspecies *paratuberculosis* infections in a dairy herd going through a control program. Prev Vet Med 2008; 83: 215–227. 17868937 10.1016/j.prevetmed.2007.07.008

[3] Momotani E, Whipple DL, Thiermann AB, Cheville NF. Role of M cells and macrophages in the entrance of *Mycobacterium paratuberculosis* into domes of ileal Peyer's patches in calves. Vet Pathol 1988;25: 131–137. 3363791 10.1177/030098588802500205

[4] Sigurthardottir OG, Press CM, Evensen O. Uptake of *Mycobacterium avium* subsp. *paratuberculosis* through the distal small intestinal mucosa in goats: An ultrastructural study. Vet Pathol 2001;38: 184–189. 11280374 10.1354/vp.38-2-184

[5] Arsenault RJ, Maattanen P, Daigle J, Potter A, Griebel P, Napper S. From mouth to macrophage: Mechanisms of innate immune subversion by *Mycobacterium avium* subsp. *paratuberculosis*. Vet Res 2014;45: 54 10.1186/1297-9716-45-54 24885748 PMC4046017

[6] Gonzalez J, Geijo MV, Garcia-Pariente C, Verna A, Corpa JM, Reyes LE, et al Histopathological classification of lesions associated with natural paratuberculosis infection in cattle. J Comp Pathol 2005;133: 184–196. 16045917 10.1016/j.jcpa.2005.04.007

[7] Koets A, Rutten V, Hoek A, van Mil F, Muller K, Bakker D, et al Progressive bovine paratuberculosis is associated with local loss of CD4+ T cells, increased frequency of T cells, and related changes in T-cell function. Infect Immun 2002;70: 3856–3864. 12065529 10.1128/IAI.70.7.3856-3864.2002 PMC128076

[8] Waters WR, Miller JM, Palmer MV, Stabel JR, Jones DE, Koistinen KA, et al Early induction of humoral and cellular immune responses during experimental *Mycobacterium avium* subsp. *paratuberculosis* infection of calves. Infect Immun 2003; 71: 5130–5138. 12933856 10.1128/IAI.71.9.5130-5138.2003 PMC187349

[9] Allen AJ, Park KT, Barrington GM, Lahmers KK, Hamilton MJ, Davis WC. Development of a bovine ileal cannulation model to study the immune response and mechanisms of pathogenesis of paratuberculosis. Clin Vaccine Immunol 2009;16: 453–463. 10.1128/CVI.00347-08 19225077 PMC2668272

[10] Wu CW, Livesey M, Schmoller SK, Manning EJ, Steinberg H, Davis WC, et al Invasion and persistence of *Mycobacterium avium* subsp. *paratuberculosis* during early stages of Johne's disease in calves. Infect Immun 2007;75: 2110–2119. 17296749 10.1128/IAI.01739-06 PMC1865790

[11] Khare S, Nunes JS, Figueiredo JF, Lawhon SD, Rossetti CA, Gull T, et al Early phase morphological lesions and transcriptional responses of bovine ileum infected with *Mycobacterium avium* subsp. *paratuberculosis*. Vet Pathol 2009;46: 717–728. 10.1354/vp.08-VP-0187-G-FL 19276052

[12] Charavaryamath C, Gonzalez-Cano P, Fries P, Gomis S, Doig K, Scruten E, et al Host responses to persistent *Mycobacterium avium* subspecies *paratuberculosis* infection in surgically isolated bovine ileal segments. Clin Vaccine Immunol 2013;20: 156–165. 10.1128/CVI.00496-12 23221000 PMC3571287

[13] Ponnusamy D, Periasamy S, Tripathi BN, Pal A. *Mycobacterium avium* subsp. *paratuberculosis* invades through M cells and enterocytes across ileal and jejunal mucosa of lambs. Res Vet Sci 2013;94: 306–312. 10.1016/j.rvsc.2012.09.023 23122809

[14] Griebel PJ, Hein WR. Expanding the role of Peyer's patches in B-cell ontogeny. Immunol Today 1996;17: 30–39. 8652050 10.1016/0167-5699(96)80566-4

[15] Mutwiri G, Watts T, Lew L, Beskorwayne T, Papp Z, Baca-Estrada ME, et al Ileal and jejunal Peyer's patches play distinct roles in mucosal immunity of sheep. Immunol 1999;97: 455–461. 10.1046/j.1365-2567.1999.00791.x PMC2326853 10447767

[16] Parsons KR, Howard CJ, Jones BV, Sopp P. Investigation of bovine gut associated lymphoid tissue (GALT) using monoclonal antibodies against bovine lymphocytes. Vet Pathol 1989;26: 396–408. 2588436 10.1177/030098588902600505

[17] Yasuda M, Fujino M, Nasu T, Murakami T. Histological studies on the ontogeny of bovine gut-associated lymphoid tissue: Appearance of T cells and development of IgG+ and IgA+ cells in lymphoid follicles. Dev Comp Immunol 2004;28: 357–369. 14698221 10.1016/j.dci.2003.09.013

[18] Liljavirta J, Ekman A, Knight JS, Pernthaner A, Iivanainen A, Niku M. Activation-induced cytidine deaminase (AID) is strongly expressed in the fetal bovine ileal Peyer's patch and spleen and is associated with expansion of the primary antibody repertoire in the absence of exogenous antigens. Mucosal Immunol 2013;6: 942–949. 10.1038/mi.2012.132 23299615

[19] Fries P, Popowych YI, Guan LL, Beskorwayne T, Potter A, Babiuk L, Griebel PJ. Mucosal dendritic cell subpopulations in the small intestine of newborn calves. Dev Comp Immunol 2011;35: 1040–1051. 10.1016/j.dci.2011.04.003 21527286

[20] Facciuolo A, Kelton DF, Mutharia LM. Novel secreted antigens of *Mycobacterium paratuberculosis* as serodiagnostic biomarkers for Johne's disease in cattle. Clin Vaccine Immunol 2013;20: 1783–1791. 10.1128/CVI.00380-13 24089453 PMC3889510

[21] Waters WR, Miller JM, Palmer MV, Stabel JR, Jones DE, Koistinen KA, et al Early induction of humoral and cellular immune responses during experimental *Mycobacterium avium* subsp. *paratuberculosis* infection of calves. Infect Immun 2003;71: 5130–5138. 12933856 10.1128/IAI.71.9.5130-5138.2003 PMC187349

[22] Lowden S, Heath T. Lymph pathways associated with Peyer's patches in sheep. J Anat 1992;181: 209–217. 1295861 PMC1259717

[23] Clarke CJ, Little D. The pathology of ovine paratuberculosis: Gross and histological changes in the intestine and other tissues. J Comp Pathol 1996;114: 419–437. 8814536 10.1016/s0021-9975(96)80017-x

[24] Yasuda M, Jenne CN, Kennedy LJ, Reynolds JD. The sheep and cattle Peyer's patch as a site of B-cell development. Vet Res 2006;37: 401–415. 16611555 10.1051/vetres:2006008

[25] Lindhout E, Koopman G, Pals ST, de Groot C. Triple check for antigen specificity of B cells during germinal centre reactions. Immunol Today 1997;18: 573–577. 9425734 10.1016/s0167-5699(97)01160-2

[26] Reynaud CA, Garcia C, Hein WR, Weill JC. Hypermutation generating the sheep immunoglobulin repertoire is an antigen-independent process. Cell 1995;80: 115–125. 7813007 10.1016/0092-8674(95)90456-5

[27] Motyka B, Reynolds JD. Apoptosis is associated with the extensive B cell death in the sheep ileal Peyer's patch and the chicken bursa of fabricius: A possible role in B cell selection. Eur J Immunol 1991;21: 1951–1958. 1868877 10.1002/eji.1830210825

[28] Fadok VA, Bratton DL, Konowal A, Freed PW, Westcott JY, Henson PM. Macrophages that have ingested apoptotic cells in vitro inhibit proinflammatory cytokine production through autocrine/paracrine mechanisms involving TGF-beta, PGE2, and PAF. J Clin Invest 1998;101: 890–898. 9466984 10.1172/JCI1112 PMC508637

[29] Abitorabi MA, Mackay CR, Jerome EH, Osorio O, Butcher EC, Erle DJ. Differential expression of homing molecules on recirculating lymphocytes from sheep gut, peripheral, and lung lymph. J Immunol 1996;156: 3111–3117. 8617931

[30] Begg DJ, de Silva K, Plain KM, Purdie AC, Dhand N, Whittington RJ. Specific faecal antibody responses in sheep infected with *Mycobacterium avium* subspecies *paratuberculosis*. Vet Immunol Immunopathol 2015;166: 125–131. 10.1016/j.vetimm.2015.06.011 26144891

[31] Hostetter J, Kagan R, Steadham E. Opsonization effects on *Mycobacterium avium* subsp. *paratuberculosis*—macrophage interactions. Clin Diagn Lab Immunol 2005;12: 793–796. 15939756 10.1128/CDLI.12.6.793-796.2005 PMC1151980

[32] Everman JL, Bermudez LE. Antibodies against invasive phenotype-specific antigens increase *Mycobacterium avium* subspecies *paratuberculosis* translocation across a polarized epithelial cell model and enhance killing by bovine macrophages. Front Cell Infect Microbiol 2015;5: 58 10.3389/fcimb.2015.00058 26301206 PMC4528203

[33] Secott TE, Lin TL, Wu CC. *Mycobacterium avium* subsp. *paratuberculosis* fibronectin attachment protein facilitates M-cell targeting and invasion through a fibronectin bridge with host integrins. Infect Immun 2004;72: 3724–3732. 15213112 10.1128/IAI.72.7.3724-3732.2004 PMC427427

[34] Bannantine JP, Bayles DO, Waters WR, Palmer MV, Stabel JR, Paustian ML. Early antibody response against *Mycobacterium avium* subspecies *paratuberculosis* antigens in subclinical cattle. Proteome Sci 2008;6: 5 10.1186/1477-5956-6-5 18226229 PMC2265687

[35] Mortier RA, Barkema HW, Negron ME, Orsel K, Wolf R, De Buck J. Antibody response early after experimental infection with *Mycobacterium avium* subspecies *paratuberculosis* in dairy calves. J Dairy Sci 2014; 97:5558–5565. 10.3168/jds.2014-8139 24996279

[36] Charavaryamath C, Fries P, Gomis S, Bell C, Doig K, Guan LL, et al Mucosal changes in a long-term bovine intestinal segment model following removal of ingesta and microflora. Gut Microbes 2011;2: 134–144. 10.4161/gmic.2.3.16483 21869606

