# Supplementary figures and images for: Marked Differences in Mucosal Immune Responses Induced in Ileal versus Jejunal Peyer’s Patches to Mycobacterium avium subsp. paratuberculosis Secreted Proteins following Targeted Enteric Infection in Young Calves
Source: PLoS One. 2016 Jul 7;11(7):e0158747. doi: 10.1371/journal.pone.0158747 (PMC4936678; doi:10.1371/journal.pone.0158747)

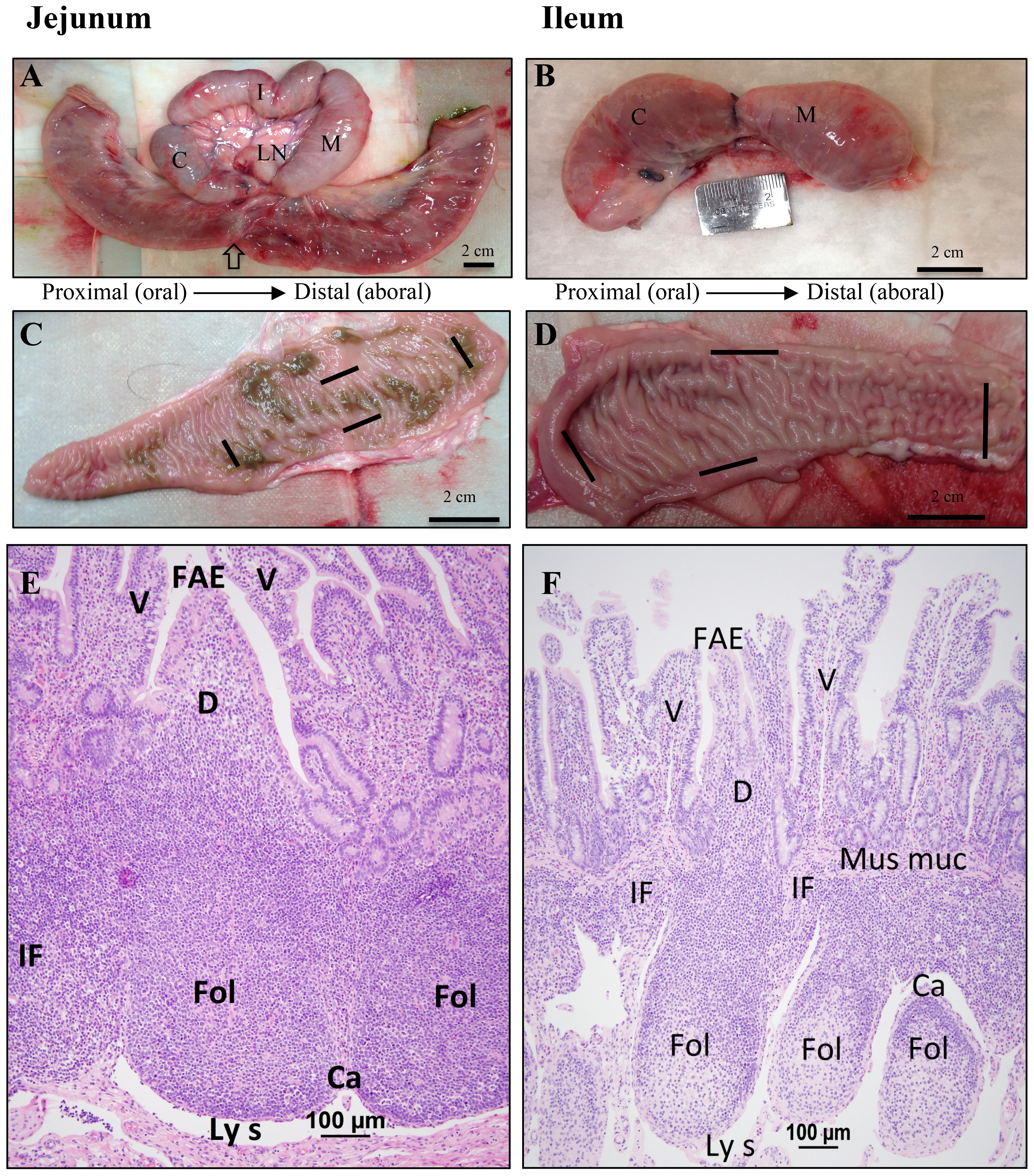

Supplement: S1 Fig — Representative gross anatomy of mid-jejunal (A) and terminal small intestine (B) segment consisting of 2 or 3 compartments: C, control compartment injected with PBS; I, interspace compartment; M, M. avium subsp. paratuberculosis-infected compartment inoculated with 109 CFUs. Arrow denotes the site of anastomosis to re-establish continuity of the intestinal tract. Representative picture of the gross appearance of the mucosal surface of discrete JPP (C) and continuous IPP (D) with PP demarcated by black lines. Hematoxylin and eosin staining of JPP (E) and IPP (F) from M. avium subsp. paratuberculosis-infected compartments. Ca, capsule; D, dome region; FAE, follicle associated epithelium; Fol, follicle; IF, interfollicular region; LN, lymph node; Lys, lymphatic sinus; Mus muc, muscularis mucosa; V, villus. (TIF) [file pone.0158747.s001.tif]

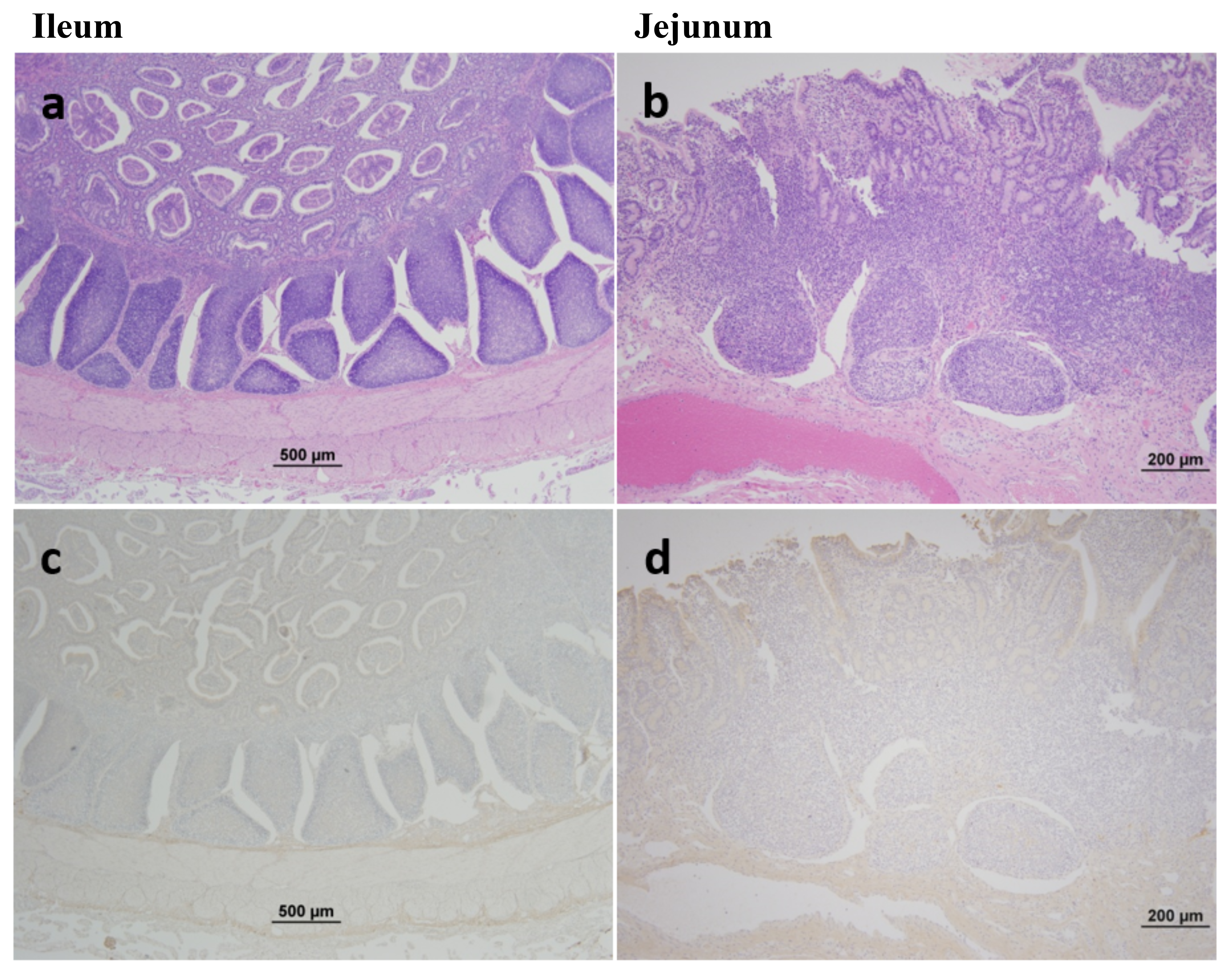

Supplement: S2 Fig — Hematoxylin and eosin stain of IPP (a) and JPP (b). Immunohistochemistry of IPP (c) and JPP (d) with rabbit anti-M. avium subsp. paratuberculosis antibodies. (TIF) [file pone.0158747.s002.tif]

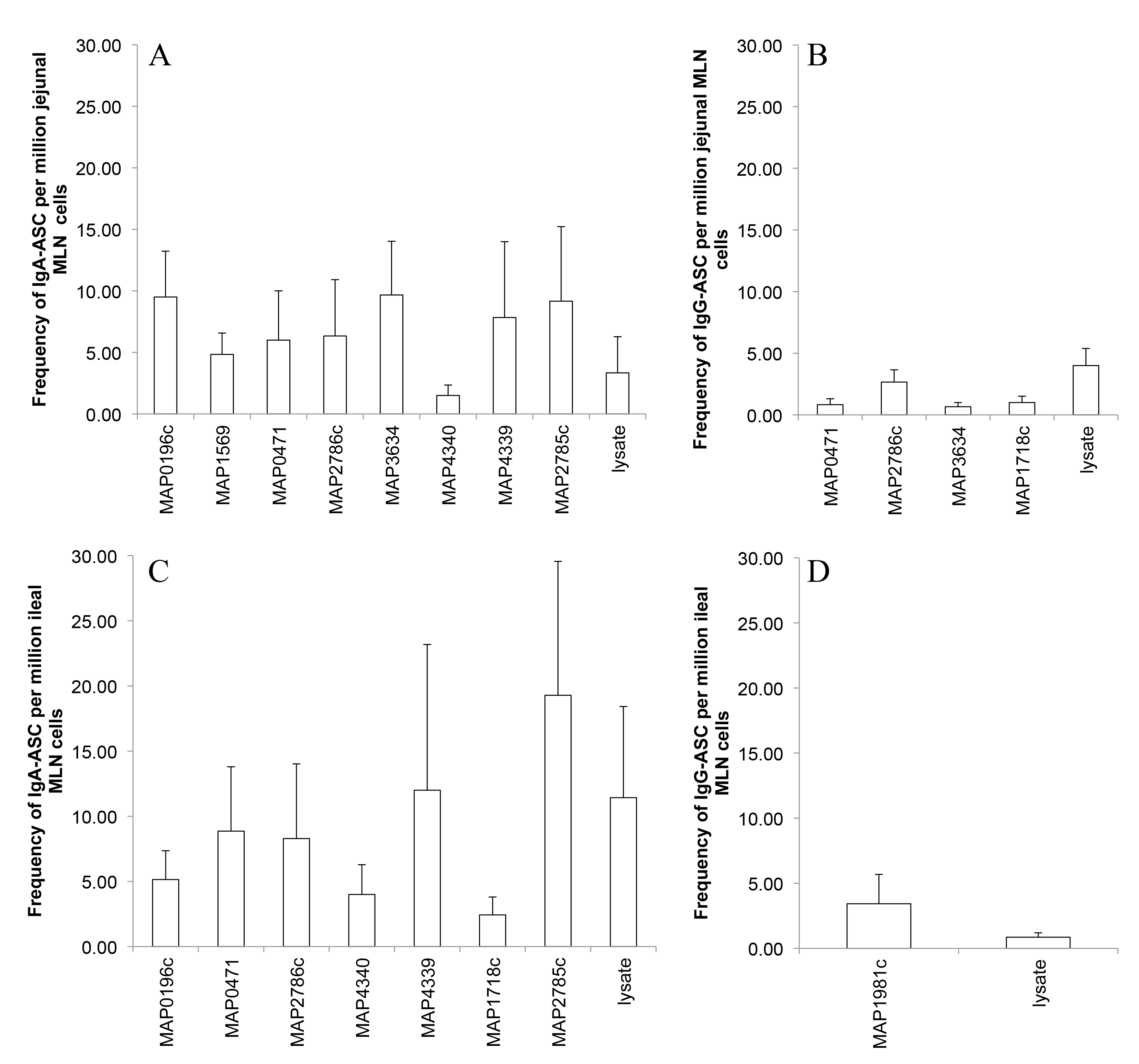

Supplement: S3 Fig — Frequency of ASCs specific for M. avium subsp. paratuberculosis recombinant proteins and lysate in cell suspensions prepared from MLNs draining M. avium subsp. paratuberculosis-infected mid-jejunal (A, B) and terminal small intestinal (C, D) segments. Mean values of IgA- (A, C) and IgG- (B, D) ASCs are presented with SEM. (TIF) [file pone.0158747.s003.tif]

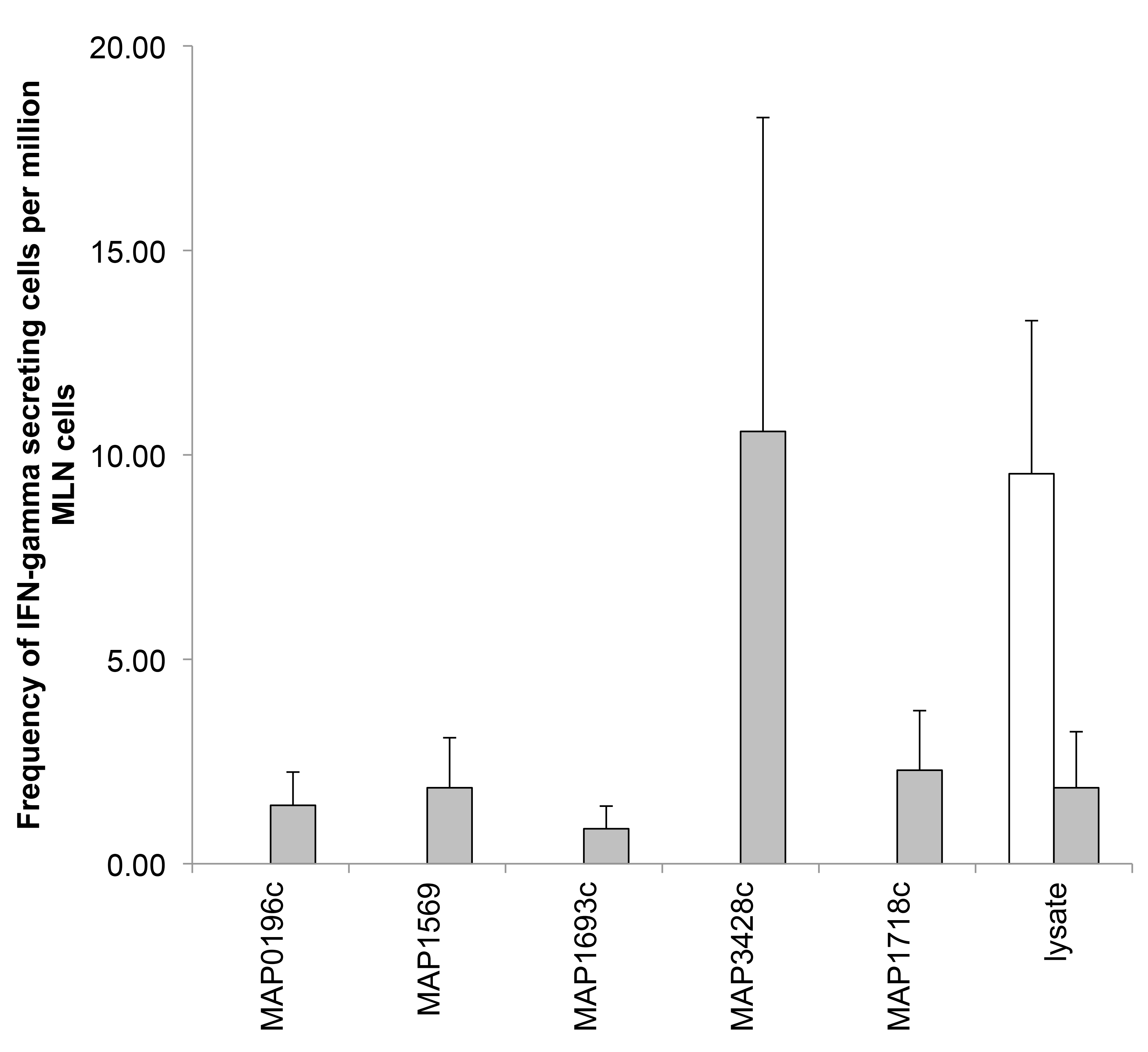

Supplement: S4 Fig — Data presented are mean values with SEM. (TIF) [file pone.0158747.s004.tif]
