# Supplementary material for: Marked Differences in Mucosal Immune Responses Induced in Ileal versus Jejunal Peyer’s Patches to Mycobacterium avium subsp. paratuberculosis Secreted Proteins following Targeted Enteric Infection in Young Calves
Source: PLoS One. 2016 Jul 7;11(7):e0158747. doi: 10.1371/journal.pone.0158747 (PMC4936678; doi:10.1371/journal.pone.0158747)
Supplement: S1 Table — (PDF) [file pone.0158747.s005.pdf]

S1 Table. Recombinant *M. avium* subsp. *paratuberculosis* proteins used to evaluate immune responses.

Protein ID	Theoretical mass kDa	Recombinant mass kDa	Annotation and putative domain(s)	Expression Plasmid
**MAP0196c**	46	61*	Septum formation	pET-30a
**MAP1569**	36	57*	FAP	pET-30a
**MAP0471**	28	35	Shisa	pET-30a
**MAP1981c**	27	27	Nucleic acid binding	pET-30a
**MAP3634**	35	38	Transpeptidase, heme binding, FAP	pET-30a
**MAP1693c**	18	18*	Peptidyl-prolyl cis-trans isomerase	pET-30a
**MBP-MAP0281**	58	61	Cysteine-rich secretory protein	pMal-c4e
**MBP-MAP3428c**	65	72	Cutinase-like protein	pMal-c4e
**MAP2785c**	17	13	DUF	pET-30a
**MAP2786c**	11	15	Rab5-binding domain	pET-30a
**MAP1718c**	15	15*	DUF	pET-30a
**MAP4340**	37	37	trxC, thioredoxin 1	pET-28a
**MAP4339**	20	24	trxB2, thioredoxin reductase	pET-30a

* Two or more protein species observed in SDS-PAGE
FAP, fibronectin-attachment protein
Shisa, eukaryotic transcription-factor type protein
MBP, maltose-binding protein
DUF, domain of unknown function
Rab, small GTPase
